# Alignathon: a competitive assessment of whole-genome alignment methods

**DOI:** 10.1101/gr.174920.114

**Published:** 2014-12

**Authors:** Dent Earl, Ngan Nguyen, Glenn Hickey, Robert S. Harris, Stephen Fitzgerald, Kathryn Beal, Igor Seledtsov, Vladimir Molodtsov, Brian J. Raney, Hiram Clawson, Jaebum Kim, Carsten Kemena, Jia-Ming Chang, Ionas Erb, Alexander Poliakov, Minmei Hou, Javier Herrero, William James Kent, Victor Solovyev, Aaron E. Darling, Jian Ma, Cedric Notredame, Michael Brudno, Inna Dubchak, David Haussler, Benedict Paten

**Affiliations:** 1 Center for Biomolecular Science and Engineering, University of California Santa Cruz, Santa Cruz, California 95064, USA;; 2 Biomolecular Engineering Department, University of California Santa Cruz, Santa Cruz, California 95064, USA;; 3 School of Computer Science, McGill University, Montreal, QC H3A 0G4, Canada;; 4 Department of Biology, The Pennsylvania State University, University Park, Pennsylvania 16801, USA;; 5 European Molecular Biology Laboratory, European Bioinformatics Institute, Wellcome Trust Genome Campus, Hinxton, Cambridge, CB10 1SD, United Kingdom;; 6 Softberry Inc., Mount Kisco, New York 10549, USA;; 7 Department of Animal Biotechnology, Konkuk University, Seoul 143-701, Korea;; 8 Centre For Genomic Regulation (CRG), 08003 Barcelona, Spain;; 9 Universitat Pompeu Fabra (UPF), 08003 Barcelona, Spain;; 10 Westfalian Wilhelms University, Institute of Evolution and Biodiversity, 48149 Muenster, Germany;; 11 Institute of Human Genetics (IGH), UPR 1142, CNRS, Montpellier, France;; 12 Department of Energy Joint Genome Institute, Walnut Creek, California 94598, USA;; 13 Department of Computer Science, Northern Illinois University, DeKalb, Illinois 60115, USA;; 14 The Genome Analysis Centre, Norwich Research Park, Norwich, NR4 7UH, United Kingdom;; 15 ithree Institute, University of Technology Sydney, NSW 2007, Australia;; 16 Department of Bioengineering and Institute for Genomic Biology, University of Illinois at Urbana-Champaign, Illinois 61801, USA;; 17 Department of Computer Science and the Donnelly Centre, University of Toronto, Toronto, ON M5S 3G4, Canada;; 18 Centre for Computational Medicine and the Genetics and Genome Biology Program, Hospital for Sick Children, Toronto, ON M5G 1X8, Canada;; 19 Lawrence Berkeley National Laboratory, Berkeley, California 94710, USA;; 20 Howard Hughes Medical Institute, Chevy Chase, Maryland 20815-6789, USA

## Abstract

Multiple sequence alignments (MSAs) are a prerequisite for a wide variety of evolutionary analyses. Published assessments and benchmark data sets for protein and, to a lesser extent, global nucleotide MSAs are available, but less effort has been made to establish benchmarks in the more general problem of whole-genome alignment (WGA). Using the same model as the successful Assemblathon competitions, we organized a competitive evaluation in which teams submitted their alignments and then assessments were performed collectively after all the submissions were received. Three data sets were used: Two were simulated and based on primate and mammalian phylogenies, and one was comprised of 20 real fly genomes. In total, 35 submissions were assessed, submitted by 10 teams using 12 different alignment pipelines. We found agreement between independent simulation-based and statistical assessments, indicating that there are substantial accuracy differences between contemporary alignment tools. We saw considerable differences in the alignment quality of differently annotated regions and found that few tools aligned the duplications analyzed. We found that many tools worked well at shorter evolutionary distances, but fewer performed competitively at longer distances. We provide all data sets, submissions, and assessment programs for further study and provide, as a resource for future benchmarking, a convenient repository of code and data for reproducing the simulation assessments.

Given a set of sequences, a multiple sequence alignment (MSA) is a partitioning of the residues in the sequences, be they amino acids or nucleotides, into related sets. Here, we are interested in the relationship of evolutionary homology. In other contexts, residues may be aligned with a different aim, as in structural alignments, where residues are aligned if located at the same point in a shared crystal structure (Kolodny et al. 2005). MSA is a fundamental problem in biological sequence analysis because it is a prerequisite for most phylogenetic and evolutionary analyses (Felsenstein 2003; Wallace et al. 2005; Edgar and Batzoglou 2006; Notredame 2007). Most MSAs are termed “global,” made of sequences assumed to be related through the mutational processes of residue substitution, subsequence insertion, and subsequence deletion (collectively, insertions and deletions are termed indels) (for review, see Notredame 2007). The availability of whole-genome sequences has led to an interest in MSAs for complete genomes, including all sequences: genes, promoters, repetitive regions, etc. Termed whole-genome alignment (WGA), this requires the aligner to additionally consider genome rearrangements, such as inversions, translocations, chromosome fusions, chromosome fissions, and reciprocal translocations. Some tools for WGA are also capable of modeling unbalanced rearrangements that lead to copy number change, such as tandem and segmental duplications (Blanchette et al. 2004; Miller et al. 2007; Paten et al. 2008, 2011; Angiuoli and Salzberg 2011). WGA methods have been critical to understanding the selective forces acting across genomes, allowing evolutionary analysis of many potential functional elements (The ENCODE Project Consortium 2012), and in particular, the identification of conserved noncoding functional elements (*Drosophila* 12 Genomes Consortium 2007; Lindblad-Toh et al. 2011), including *cis*-regulatory elements (Kellis et al. 2003), enhancers, and noncoding RNAs.

The lack of accepted gold standard reference alignments has made it hard to objectively assess the relative merits of WGA methods. Previous evaluations of MSAs can be split into roughly four types: those using simulation, those using expert information, those using direct statistical assessments, and finally those that assess how well an alignment functions for a downstream analysis. We briefly describe and review these approaches (for a more comprehensive review, see Iantorno et al. 2014).

In simulation evaluations, a set of sequences and an alignment is generated using a model of evolution. Alignments are created from the simulated sequences and the resulting predictions are compared to the “true” simulated alignment. There are two basic types of simulators for DNA sequence evolution: coalescent simulators and noncoalescent forward-time simulators (Carvajal-Rodríguez 2010). Although useful for modeling populations, coalescent simulators cannot yet efficiently model general sequence evolution, and as a result MSA simulators currently use forward-time approaches. There are numerous forward-time simulators useful for assessing global MSA tools (Stoye et al. 1997; Blanchette et al. 2004; Cartwright 2005; Varadarajan et al. 2008). However, the simulation options for assessing WGA have until recently been absent, essentially because to do so requires modeling both low-level sequence evolution and higher-level genome rearrangements—a formidable challenge given the large and complex parameter space that potentially encompasses all aspects of genome evolution. The sgEvolver simulator (Darling et al. 2004, 2010) is used to generate simulated genome alignments, although it lacks an explicit model for sequence translocation or mobile element evolution. EvolSimulator is a genome simulator, but it has a somewhat simple model of evolution and a focus on ecological parameters (Beiko and Charlebois 2007). Another option, the ALF simulator (Dalquen et al. 2012), models gene and neutral DNA evolution. For this study we used the EVOLVER software, which can simulate full-sized, multichromosome genome evolution in forward time (Edgar et al. 2009). EVOLVER models an explicitly haploid genome and lacks a population model; its framework and expert-curated extensive parameter set are intended to produce “reference-like” genomes, i.e., haploid genomes. EVOLVER models DNA sequence evolution with sequence annotations; a gene model; a base-level evolutionary constraint model; chromosome evolution, including inter- and intrachromosomal rearrangements; tandem and segmental duplications; and mobile element insertions, movements, and evolution.

An alternative approach to assessing MSA is to use expert biological information not available to the aligner. Although interpreting the results of simulations is made difficult by the uncertainty to which they approximate reality, the clear advantage of using expert information is that it can be used to assess alignments of actual biological sequences. For protein and RNA alignment there are several popular benchmarks that provide either reference structural alignments or expertly curated alignments (Blackshields et al. 2006; Wilm et al. 2006; Kemena et al. 2013). Nontranscribed DNA alignments are, however, much harder to assess since one lacks an external criterion to assemble objective gold standard references (Kemena and Notredame 2009). This explains why untranslated DNA alignments are usually evaluated using more ad hoc expert information (Margulies et al. 2007; Paten et al. 2008). The main strength of these procedures is that they provide an objective evolutionary context when evaluating the alignment. The difficulty with relying upon such expert information is that it may address only a small fraction of the alignment (e.g., in the referenced papers, coding exons, and ancient repeats), may itself rely on other forms of inference (e.g., ancient repeat analyses have an explicit dependence on the sequence alignment procedures used to determine ancestral repeat relationships), and have unknown variance, generality, and discriminative power.

The third approach addresses alignments by statistical measures. For global MSA there are several options, e.g., the T-Coffee CORE/TCS index (Notredame and Abergel 2003; Chang et al. 2014), Heads or Tails (HoT) (Landan and Graur 2008), GUIDANCE (Penn et al. 2010a,b), and StatSigMA-w (Chen and Tompa 2010). For this work, we expand on the probabilistic sampling-based alignment reliability (PSAR) (Kim and Ma 2011) method, which samples pairwise suboptimal alignments to assess the reliability of MSAs. Statistical measures are attractive because they can be used with the complete alignments of real sequences. However, without a gold standard to compare against, they are only a proxy to a true assessment of accuracy.

The final category of common assessment methods addresses how well a program generates alignments for a given computational task. This is typically the assessment made by a biologist in choosing an alignment program, i.e., how well does it perform in practice, according to intuition or analysis? Unfortunately, these assessments, often being one-offs, rarely make it into the literature and are difficult if not impossible to generalize from because these assessments are made for the purposes of a given analysis. Notably for WGAs, Bradley et al. (2009) assessed how much alignment methods influenced de novo ncRNA predictions and Margulies et al. (2007) analyzed the effect of different WGAs on the prediction of conserved elements.

There have been relatively few independent or community organized assessments of WGA pipelines. Notably, as part of the ENCODE Pilot Project (Margulies et al. 2007), four pipelines were assessed across a substantial number of regions, and Chen and Tompa later compared those alignments using the StatSigMA-w tool (Chen and Tompa 2010). The Alignathon is an attempt to perform a larger and more comprehensive evaluation. It is a natural intellectual successor to the Assemblathon collaborative competitions (Earl et al. 2011; Bradnam et al. 2013). The starting point of the Alignathon is to assume that the problem of genome assembly is largely a solved problem. Although we admit this is currently a dubious assumption, it appears that the problem of genome assembly will shrink in size in the coming years as new sequencing technologies become available and existing assembly software is perfected to take advantage of more numerous, longer, and less error-prone reads (Branton et al. 2008; Schreiber et al. 2013; Laszlo et al. 2014). With this future as a starting point, the question a biologist faces changes from a proximate one of “how do I best assemble the genome of my favorite species?” to a higher level question of “how is my favorite species related to the pantheon of other sequenced species?” Such a question is answered through a WGA. If organized community efforts to sequence large numbers of genomes, such as the Genome 10K Project for vertebrates and 5000 arthropod genomes initiative (i5K) for insects, are to maximally fulfill their promise by revealing and refining the evolutionary history of all of their species, then it is vital that we have the best possible methods for WGA (Genome 10K Community of Scientists 2009; i5K Consortium 2013).

## Results

Of the four discussed strategies to assess alignments we pursue two: simulations and statistical assessment. We now describe the Alignathon data sets, the submissions we received, how the submissions were processed, and the evaluations that were performed.

### Data sets

**Figure 1.**
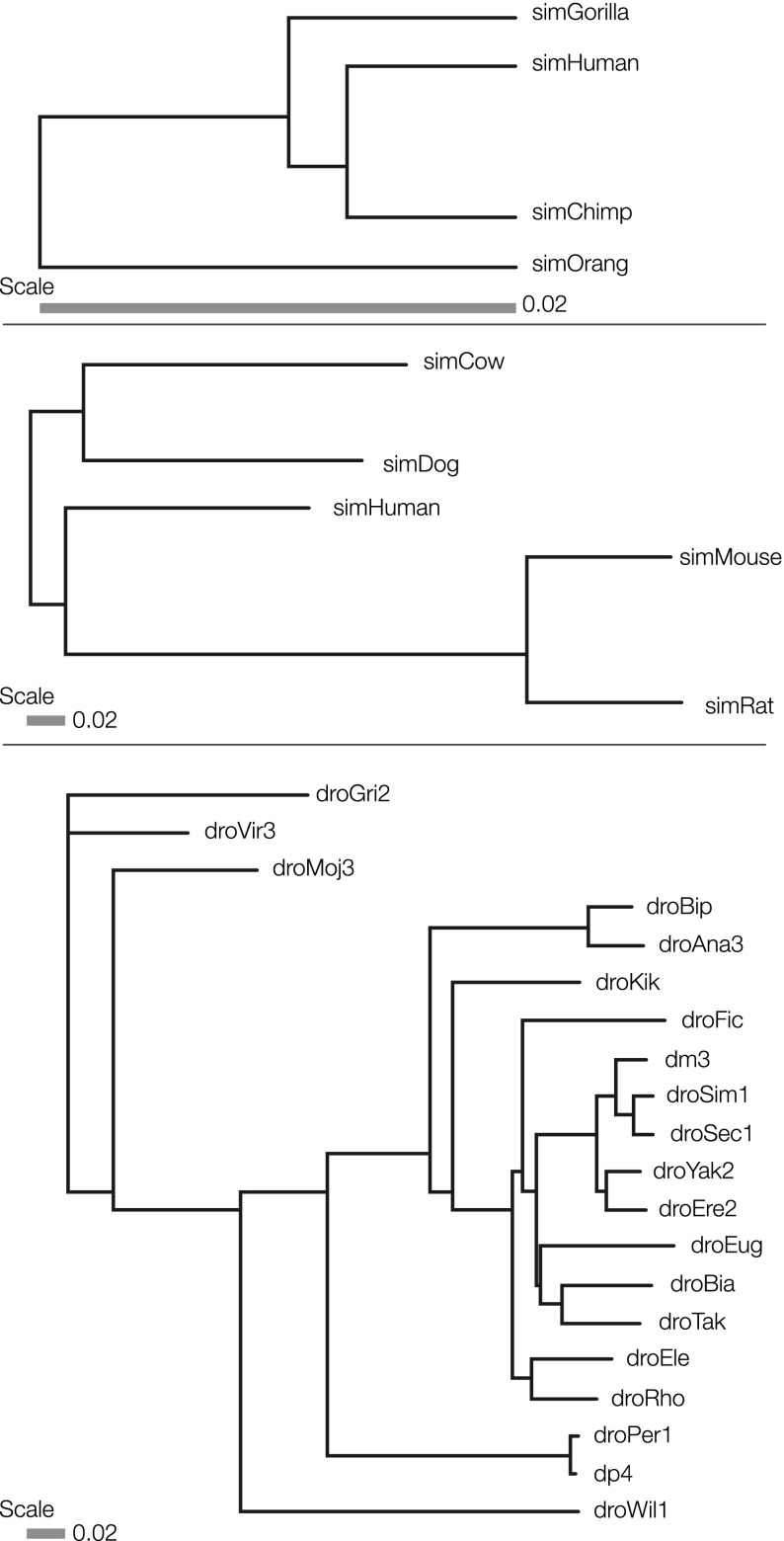
The phylogenies of the three test sets: primate simulation, mammal simulation, and real fly data set. Branch lengths are in units of neutral substitutions per site.

The Alignathon used three test sets. Two of the test sets were created by way of forward-time simulation, using the EVOLVER tool, starting from a ∼1/20th scale mammalian genome, a genome size of 120 megabases (Mb), based upon a subset of hg19/GRCh37 (chromosomes 20, 21, and 22) (see Methods). The first simulated data set models a great ape phylogeny consisting of genomes with the same evolutionary relationships as humans, chimpanzees, gorillas, and orangutans (Fig. 1). The second simulated data set is based upon a mammalian phylogeny consisting of genomes with the same evolutionary relationships as humans, mice, rats, cows, and dogs (Fig. 1). On a gross level, the summary statistics of the two simulated data sets are shown in Table 1 and Supplemental Table S1. After an initial burn-in phase to shuffle the original input sequences and ensure the simulation had reached stationarity (see Methods), the primate phylogeny contained, among other changes, one chromosomal fusion and more than three million substitutions in the lineage from the most recent common ancestor (MRCA) to the simulated human. The mammal phylogeny contained, among other changes, two chromosomal splits, one fusion, and more than 27 million substitutions in the lineage from the MRCA to the simulated human.

**Table 1.**
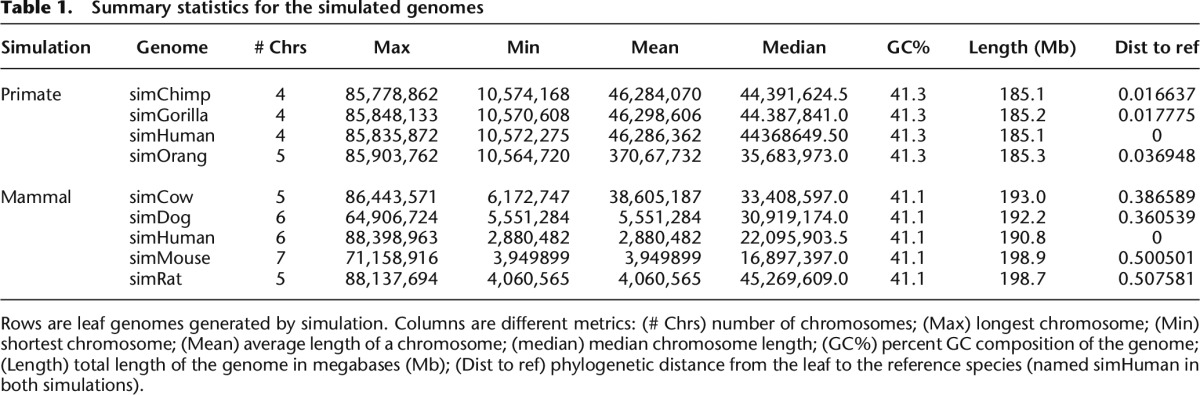
Summary statistics for the simulated genomes

Recognizing the limitations of simulations, our third test set consisted of 20 real fly genomes (Fig. 1). The fly genomes were available in various states of completion from near-finished in the case of *Drosophila melanogaster* (dm3 assembly, chromosome sequences) to fragmentary in the case of *D. rhopaloa* (droRho assembly, 34,000 contigs) (Table 2).

**Table 2.**
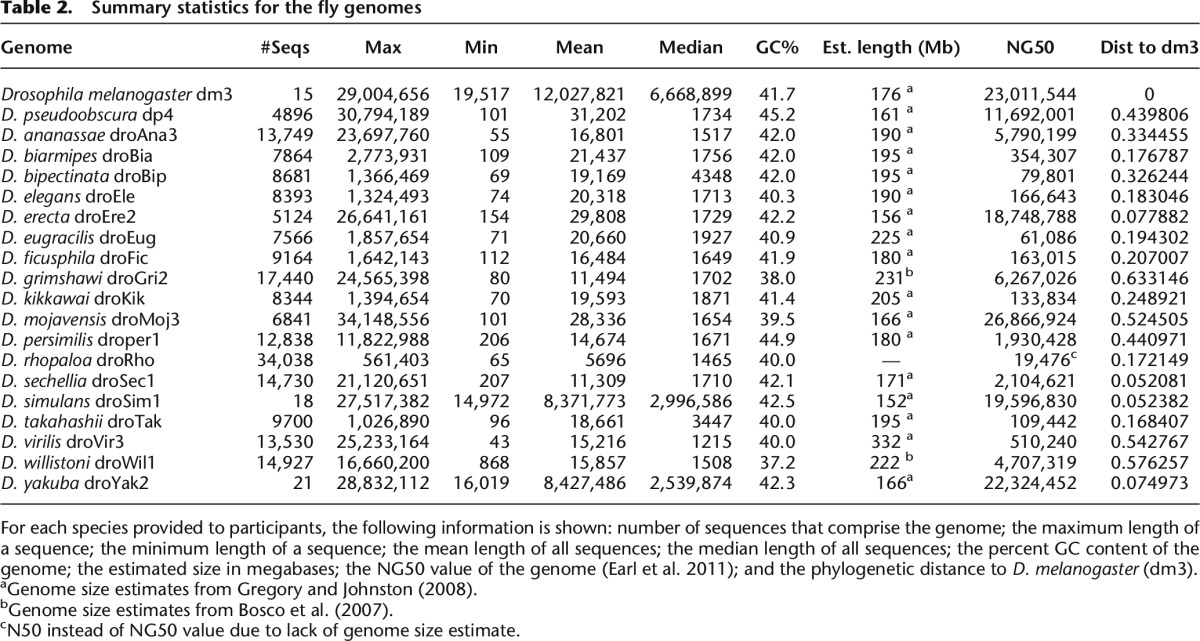
Summary statistics for the fly genomes

### Competition organization and submissions

The initial data sets were released in December 2011 and teams were given until February 2012 to submit their entries. The initial simulated data sets included the truths and information on where to obtain (and optionally contribute to) the analysis software. As in the Assemblathons, none of the teams had access to the data sets until their initial release. The Alignathon received 35 submissions, 13 for the primate simulation, 13 for the mammal simulation, and nine for the fly data set (Table 3; Supplemental Tables S2, S3, and S4). The pipelines that were used to generate the alignments represent those used by genome browsers to generate their WGAs: VISTA-LAGAN for the VISTA Browser (Frazer et al. 2004; Dubchak et al. 2009); MULTIZ for the UCSC Genome Browser (Miller et al. 2007; Meyer et al. 2013), and Pecan and EPO for the Ensembl Browser (Paten et al. 2008, 2009; Flicek et al. 2013). In addition, we tested a fairly broad set of standalone WGA tools, including progressiveMauve (Darling et al. 2010); TBA (Blanchette et al. 2004); Cactus (Paten et al. 2011); Mugsy (Angiuoli and Salzberg 2011), which was designed for closely related genomes; a meta-WGA tool, Robusta (Notredame 2012), which combines results from multiple standalone tools; and a realignment tool, PSAR-Align (Kim and Ma 2014), which was used to realign MULTIZ based alignments in this competition but can in principle refine alignments from any multiple alignment tool. We also tested pairwise WGAs from the GenomeMatch team. As might be expected, not all algorithms/pipelines were run for all test sets. Participants cited limitations of the methods applied (e.g., inability to handle the scale of the fly data set) and of resources (time, person-hours, funding, etc.) as reasons for not participating in all data sets. Descriptions generated by the teams of the computation of each submission are given in the Supplemental Material, as are details on runtimes and computational resources used.

**Table 3.**
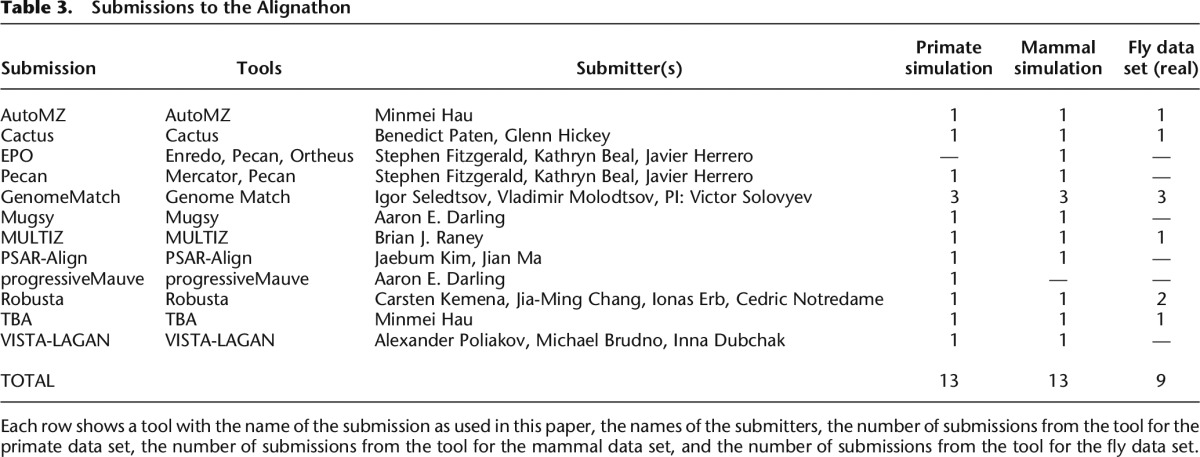
Submissions to the Alignathon

### Genome-wide comparison to simulated genome alignments

All submissions were received in multiple alignment format (MAF) (http://genome.ucsc.edu/FAQ/FAQformat.html#format5). A suite of MAF comparison tools was developed for the project (mafTools) (see Methods), including a comparator tool, so-called because it compares two alignment files. We call the set of aligned pairs of residues within an alignment its *alignment relation*. The comparator tool works by taking two input MAF files, A and B, and comparing their alignment relations. For the simulated data sets, if A is the predicted alignment created by a tool and B is the simulated truth, then the ratio of the number of pairs in the intersection of A and B to the number of pairs in A is the *precision* of the prediction. Conversely, the ratio of the number of pairs in the intersection of A and B to the number of pairs in B is the *recall* of the prediction. One standard method for combining precision and recall into a single value is the balanced F-score, which is simply the harmonic mean of precision and recall (Beitzel 2006):
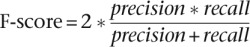


The cardinality of the alignment relation of the considered WGAs is exceedingly large, e.g., ∼1.7 billion pairs for the simulated mammalian alignment. This made complete comparison impractical. Instead, for each pair of MAFs compared, we sampled (see Methods) a subset of the alignment relation of one and checked if any or all elements of the subset were present in the alignment relation of the other. Ten million pairs were sampled for each direction of a MAF pair comparison, and variance between sampling runs was negligible (data not shown).

**Figure 2.**
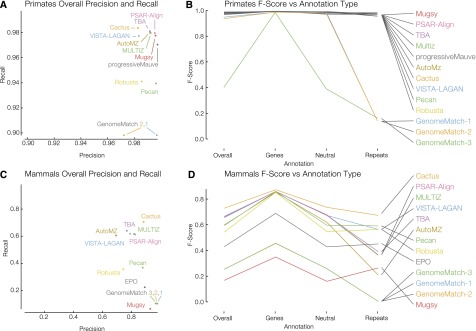
Simulated primate and mammal F-score results. Recall as a function of precision is shown for primates (*A*) and mammals (*C*). GenomeMatch-3 is omitted from plot *A* because both of its values are low (see its overall F-score in *B*). (*B*) The primate F-score results isolated to different annotation types: overall, genes, neutral and repetitive regions. (*D*) The mammal version of *C*. Legends for *B* and *D* are ordered as in the overall category and this order is maintained in the genes and neutral annotations.

For the simulated data sets we performed analyses both with respect to the entire genome and to areas of the genome subsetted by annotation type (genic, neutral, and repetitive) (see Methods). Results are shown in Figure 2 and Supplemental Tables S5–S8. We find that many of the submissions were able to align the primate data set with both relatively high recall and precision, and with the exception of the GenomeMatch submissions, which had lower values in the repetitive regions, the performance was consistently high across annotation types, e.g., the top eight submissions differed by only 0.007 in F-score and all had recall and precision above 0.98.

For the mammal simulations we found a much wider spread of results, both between aligners and within different annotation classes. The strongest submission, Cactus, had an F-score 0.081 points higher than its nearest competitor. Looking at the mammal results by annotation type, generally (and predictably) submissions performed the best in genic regions, where simulated selection, which led to strong conservation, was presumably highest. Performance was intermediate in neutral regions and submissions generally performed most poorly in repetitive regions. Generally, submissions retained their ranking across annotation regions, that is to say, the submissions ranked 1 and 2 overall were also ranked 1 and 2 in genic regions. However, this trend did not hold for repetitive regions; and surprisingly, several submissions performed slightly better in repetitive regions than in the neutral regions (Mugsy, Pecan, EPO, Robusta).

**Figure 3.**
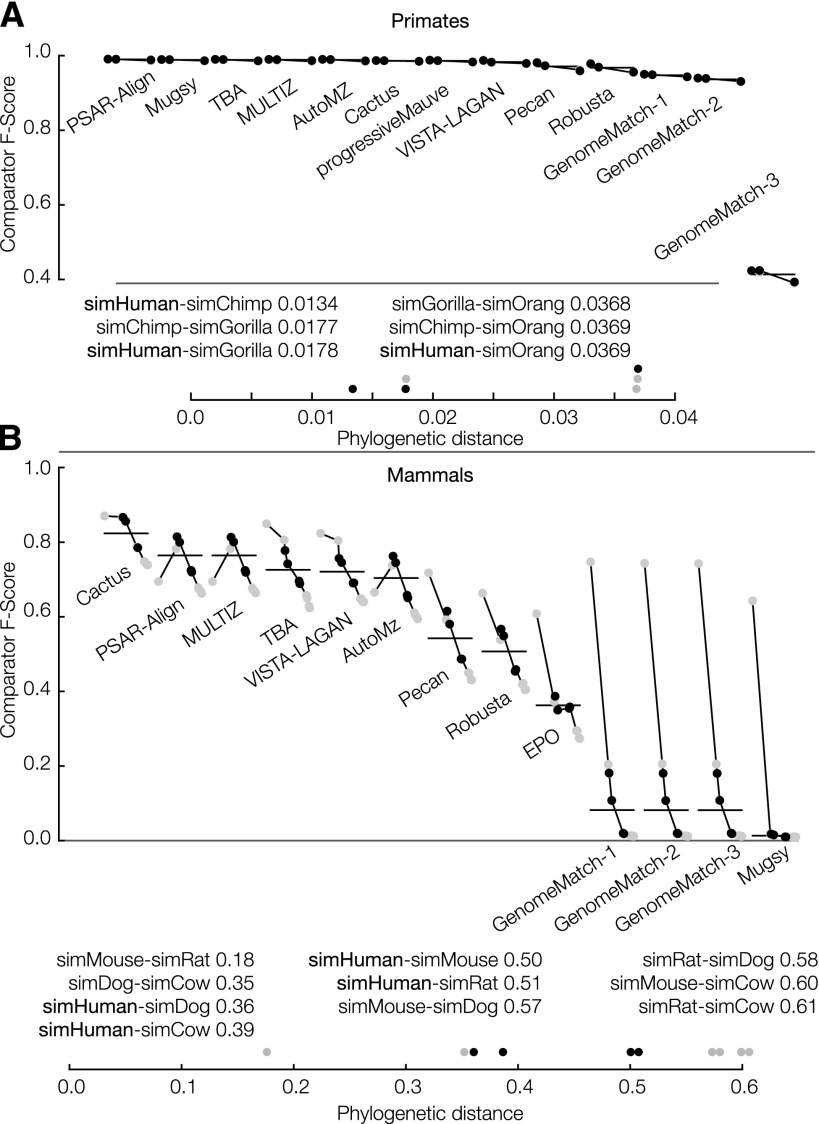
Primate (*A*) and mammal (*B*) simulation F-score results stratified by phylogenetic distance. For each subplot, the vertical axis shows the F-score and the horizontal axis shows 13 individual submissions ordered from *left* to *right* (descending) by average overall F-score. Horizontal gray lines show the overall F-score of the submission, taking into account all sequence pairs. Horizontal black lines show the overall F-score of the submission, taking into account only sequence pairs including the reference. Submissions are comprised of points connected by a line where the points are in ascending order of phylogenetic distance (all possible pairs are shown).

As phylogenetic distance between species grows the number of unobserved mutation events increases, and the alignment problem naturally becomes more difficult (Holmes and Durbin 1998; Landan and Graur 2008; Wong et al. 2008). To see this, we stratified the results by phylogenetic distance (path length between leaves in the simulated phylogenies) between all pairs of species (see Fig. 3). Longer distances are indeed observed to lead to lower precision and recall values, and therefore lower F-score values. For reference-based aligners, which use one species as a reference (here simHuman), there is a clear dip in performance for nonreference pairs (pairs not including the reference sequence). This is especially prevalent in Figure 3B for the PSAR-Align submission, which used the MULTIZ program, and for the MULTIZ and AutoMZ submissions, which also rely upon the MULTIZ program.

### Evaluating genome alignments in the absence of a true alignment

We used the recently developed PSAR statistical alignment tool (Kim and Ma 2011) to compare to the simulation results and to assess the fly data set. PSAR assesses an alignment by removing a sequence, sampling suboptimal alignments between the removed sequence and the remaining alignment using the forward algorithm with a pairwise hidden Markov model (pair-HMM), and then checking to see how well the newly sampled alignments match the original alignment. By repeatedly performing this sampling with every possible sequence, PSAR is able to calculate an alignment reliability score, termed a *PSAR pair score,* for every pair of matched residues in the alignment. Each *PSAR pair score* is similar to the posterior probability that a given pair of residues in the input alignment are aligned (Durbin et al. 1998), i.e., it can be thought of as a proxy to a local measure of accuracy that factors in the edit matrix surrounding the pair of aligned residues.

To deal with its limited alignment model—which is appropriate for global MSA, allowing only substitutions, insertions, and deletions—and to make it computationally feasible to assess the alignments, we ran PSAR on subsections of the data sets. For each of the data sets we ran PSAR on five sampled half-megabase subregions (see Methods). Subregion alignments were converted to make them appropriate for PSAR (e.g., removing duplications, ordering rows, etc.) (see Methods). For a pair of genomes we define the *PSAR-precision* as the average of the PSAR pair scores of their aligned residues. The overall *PSAR-precision* for the complete alignment is the average of PSAR-precision for genome pairs including the reference. The PSAR-precision scores are analogous to the precision measures calculated from the simulations, because they estimate the expected number of pairs in the alignment that are correctly aligned.

To complement our proxy to precision we used a simple proxy to recall: coverage. For a pair of genomes A and B, the proportion of residues in A aligned to a residue from B is the *coverage of B on A.* The *overall coverage* (where we drop the “overall” when it is clear from the context) is the average of coverages for all pairs of distinct species. Hypothesizing that PSAR-precision can be used to approximate precision and that coverage can be used as an estimate of recall, the natural statistical analog to F-score is the harmonic mean of PSAR-precision and coverage, which we call the *pseudo F-score.*

**Figure 4.**
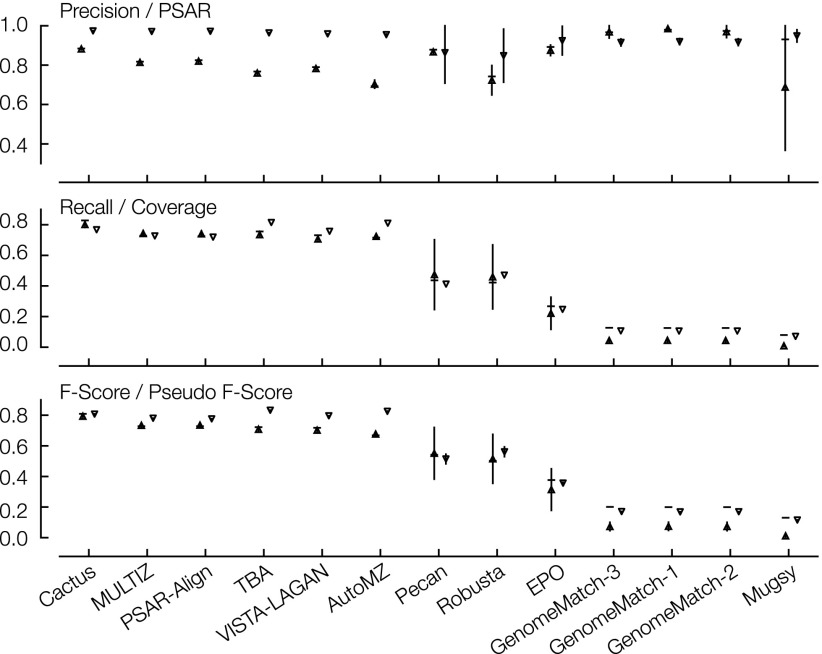
Simulated mammal results comparing simulation values to statistical values. Shown are precision and PSAR; recall and coverage; F-score and pseudo F-score. Each column represents the results of one submission; columns are in descending order of overall (full genome) F-score value. The horizontal line is, respectively, the overall precision, recall, or F-score value; the upward triangle with a vertical line is the regional precision, recall, or F-score mean value, ± the regional standard deviation; the downward triangle with a vertical line is the PSAR-precision, coverage, or pseudo F-score mean value, ± standard deviation for values that were computed using regional subalignments.

To see how consistent our statistical measures were to the measures derived from the simulations, we calculated them for the user-generated simulated primate and mammal alignments (Fig. 4; Supplemental Tables S5, S6). To check for any bias created by the use of a set of adjusted regional alignments in calculating the PSAR-precision and coverages values, we calculated regional precision and recall values using the regional alignments (see Methods) and found good correlations between the regional and overall versions of these numbers (see Methods; Fig. 4; Supplemental Tables S5, S6).

In the simulated primates all the values were uniformly high, and hence saturated. However, looking at the simulated mammals, we find a very good, linear correlation between recall and coverage (*r*^*2*^ = 0.984), but no linear correlation between PSAR-precision and precision. In particular, PSAR reports relatively consistent, high scores for all the different alignment programs, suggesting that at a local, residue level, on aggregate the alignments look equivalently reasonable between alignment programs. Despite the lack of linear correlation between precision and PSAR-precision, we find that because of the excellent recall and coverage correlation, the F-score and pseudo F-score results linearly correlate strongly (*r*^*2*^ = 0.975 in simulated mammals). This appears to be because the more limiting factor in many of the alignments’ performance was not precision, but rather a lack of relative recall/coverage, something particularly affecting the GenomeMatch, Mugsy, and to a lesser extent, Pecan, EPO, and Robusta submissions.

**Figure 5.**
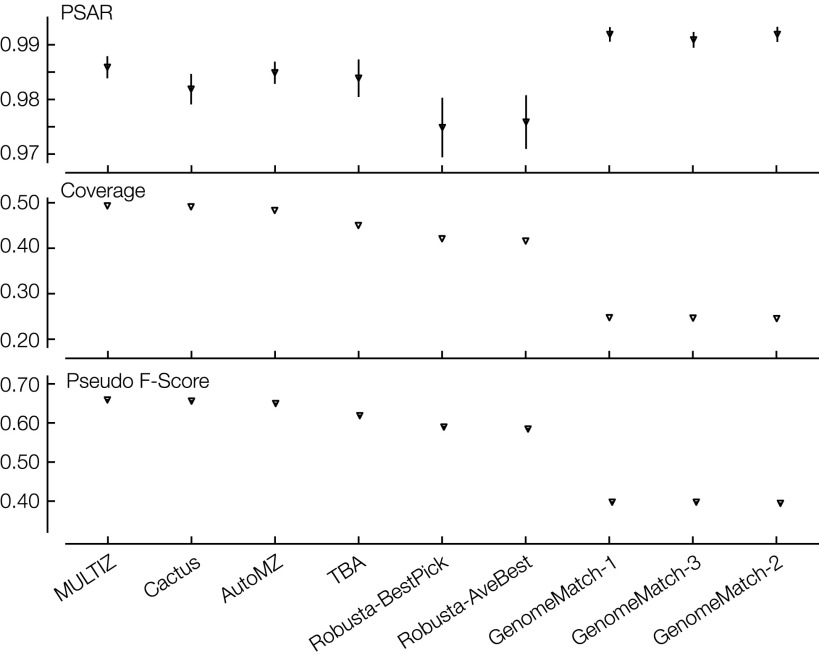
Fly results: values of PSAR-precision; average overall coverage between all pairs; pseudo F-score. Columns are in descending order of mean pseudo F-score value. For each metric, each submission is made up of a downward triangle with a vertical line representing the regional mean ± SD.

**Figure 6.**
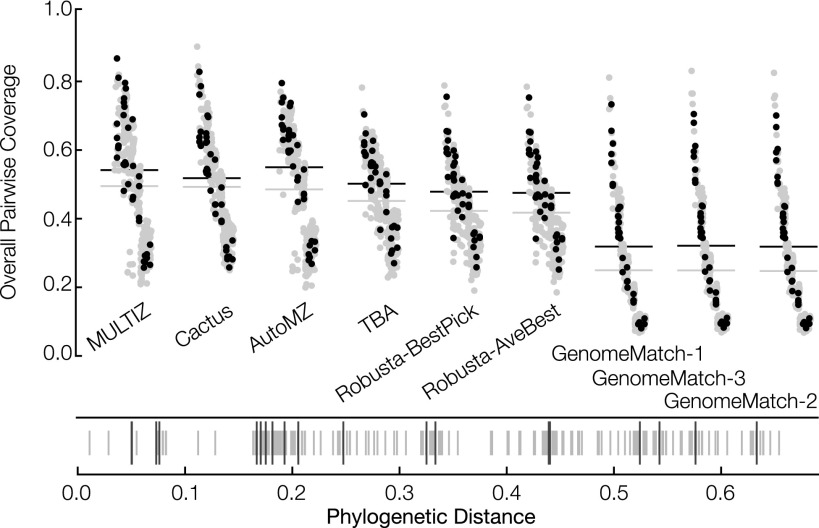
Overall pairwise coverage values in the fly data set. Submissions are ordered *left* to *right* (descending) by overall coverage. Gray points are nonreference pairs, and black points contain the reference. The horizontal gray line shows the average coverage of the submission for all points, and the horizontal black line shows the average coverage of the submission just for pairs containing the reference. *Beneath* the pairwise coverage plot is a barcode plot showing the phylogenetic distances of all pairs. Shorter gray lines are nonreference pairs and longer black lines are reference-containing pairs.

Figure 5 and Supplemental Table S9 show the overall PSAR-precision, coverage, and pseudo F-score results for the fly data set, and Supplemental Figure S3 shows the pseudo F-score stratified by phylogenetic distance for the fly data set. For the teams that submitted alignments for both data sets, we see good concordance between the fly and simulated results. Again, the difference between the aligners is dominated by coverage, with uniformly high (all greater than 0.97) average PSAR-precision values that mostly lie within the regional standard deviations of one another, with the exception of the GenomeMatch alignments, which have very high PSAR-precision values but relatively low coverage. Surprisingly, given their reference assisted nature, we find that, along with Cactus and TBA, MULTIZ and AutoMZ had high relative coverage and pseudo F-scores, even when factoring that coverage was calculated over all pairs, not just reference-containing pairs. Plotting the pairwise coverages between all pairs of species (Fig. 6), we see that all the programs had higher relative coverage for pairs involving the reference; partially, this is an artifact of the structure of the phylogeny (Fig. 1). The reference-based aligners (here MULTIZ and AutoMZ) indeed did have the highest coverage for reference pairs, and the strongest nonreference-based aligners by these metrics, TBA and Cactus, showed a smaller separation between reference and nonreference species pairs.

### Visualizing and analyzing regional accuracy

**Figure 7.**
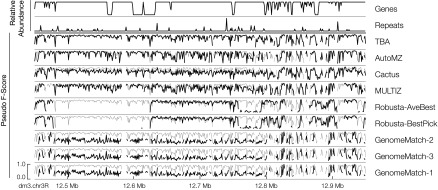
Region 2 of *D. melanogaster* (dm3) with respect to *D. grimshawi* (droGri2) of the regional analysis of the mammal simulation data set. Region 2 is defined as bases 12,450,223–12,950,222 of dm3 chromosome 3R (horizontal axis). Rows are as follows: the relative abundance of genes within the region; the relative abundance of repetitive sequence in the region; and submissions in descending order of average pseudo F-score. Each submission row shows the pseudo F-score of the submission in black. The vertical axis of each row uses the same scale as shown in the *bottom* row. The pseudo F-score value of the top submission for this region (TBA) is shown in gray in the background.

We have demonstrated that the performance of alignments varied regionally according to the simulation of annotation types. Having developed scoring metrics that can be applied across simulated and nonsimulated genomes, we corroborated this analysis by visualizing how the scores vary across the sampled subregions. To view a complete subregion at approximately this level of resolution, we binned the reference sequence into 1-kb nonoverlapping intervals and calculated the F-score (for the simulated data sets) and pseudo F-score for each bin, calculating the score for a bin as if it represented the complete alignment, and for the simulated comparison, restricting the true alignment to just those pairs involving residues in the reference interval that defined the bin. Figure 7 and Supplemental Figures S1 and S2 visualize how the scores vary across example subregions of, respectively, the simulated mammalian, real fly, and simulated primate alignments. It is clear that the “best” alignments by these measures differ substantially from the poorest, and that for many submissions there is considerable regional variation. Looking across all the simulated regions, the F-score and pseudo F-score measures correlate reasonably bin-by-bin (Supplemental Figs. S4–S6) (*r*^*2*^ = 0.671), indicating that pseudo F-score can be used as a reasonable proxy to F-score at this regional level of resolution (e.g., see Supplemental Fig. S7, the equivalent to Supplemental Fig. S2, but using pseudo F-score instead of F-score). It should be noted that the correlation is imperfect; in particular, it appears that the pseudo F-scores saturate at high values, whereas the corresponding F-scores still discriminate alignment quality, i.e., pseudo F-scores do not always discriminate between good and very good alignments.

### Comparing the submissions directly

**Figure 8.**
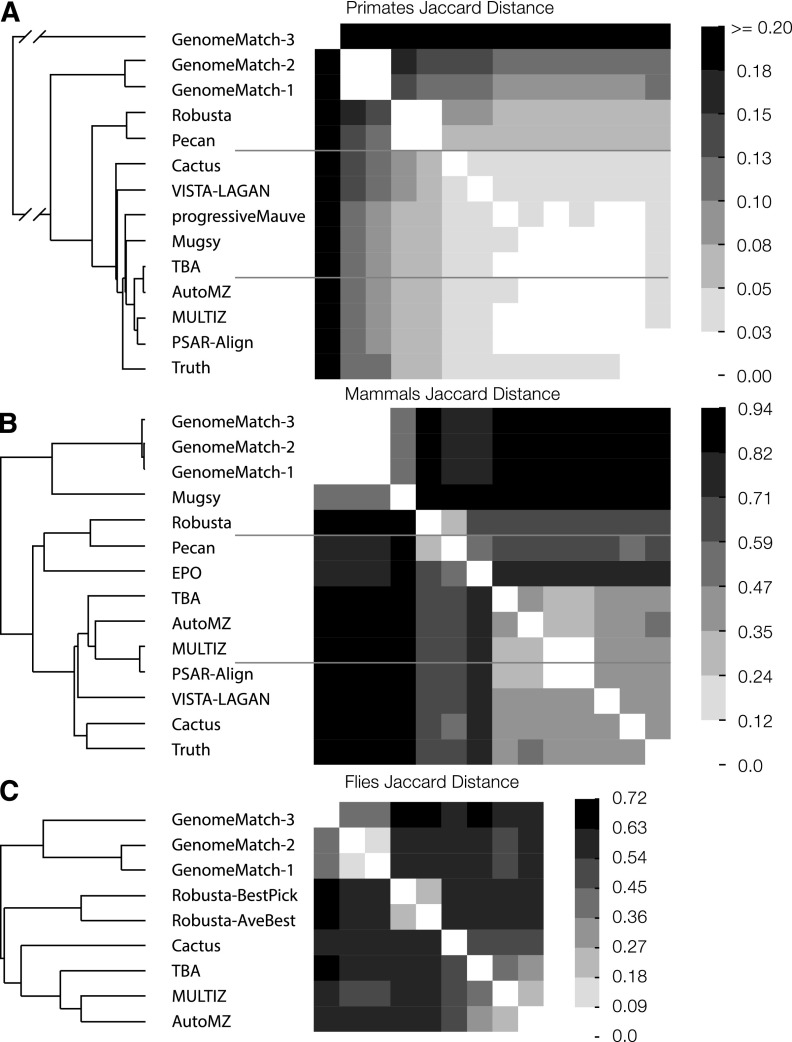
The Jaccard distance (1 − Jaccard similarity coefficient) matrix and accompanying hierarchical clustering (UPGMA) of submissions for each of the three test sets. (*A*) Primate Jaccard distance; (*B*) mammal Jaccard distance; (*C*) fly Jaccard distance. Higher values indicate that the sets of aligned pairs of two submissions are more dissimilar, and lower values indicate similarity.

Several of the pipelines used some of the same underlying programs. To see how these commonalities affected the alignments, for each data set we calculated the Jaccard distance between the alignment relations of each of the submissions (Fig. 8). As predicted by the earlier analyses, the primate submissions are relatively similar to one another, whereas the mammalian and fly submissions prove much more divergent. The inter-data set commonality between some submissions is striking, with the same patterns being repeated across the three data sets, and fits well with the programmatic commonalities that the pipelines share. The results indicate that some of the programmatic commonalities between the alignment pipelines are perhaps more important than others. For example, sharing the same synteny block generator (Mercator [Dewey 2007] or MULTIZ) appears to have had a greater effect on the results than sharing the same synteny block aligner. In particular, the EPO and Pecan submissions both use the Pecan program (Paten et al. 2008; Paten et al. 2009) to align sets of syntenic sequences, and the Cactus program (Paten et al. 2011) uses the same pairwise-HMM to generate much of its multiple alignment as Pecan, but these submissions were relatively different from one another.

### Assessing similarity versus homology

The MAF specification allows sequence residues to appear in one or more columns of the file. This allows a program to report sequence similarity, which is not a transitive property, but allows inconsistency if the intention is to report homology. For example, if a residue *x* is aligned to a residue *y* and *y* is aligned to a residue *z*, then *x* and *z* should be aligned, because it is not possible for *x* and *y* to share a common ancestor and for *y* and *z* to share a common ancestor, whereas *x* and *z* do not share a common ancestor. Comparing two alignments, one of which is transitively closed and one of which is not, based only on the aligned pairs contained in a MAF potentially gives an advantage to the nontransitively closed submission. This is because the transitively closed submission must align all residues transitively connected by alignments, which may lower the overall precision of the set of aligned pairs. To see how different the results would be if we were to have enforced transitive closure—and therefore a strict homology assumption—we created a tool (mafTransitiveClosure) that computes the transitive closure of a MAF (a linear time operation). Supplemental Figure S8 shows the results for the simulated data sets. We found that the progressiveMauve, Cactus, Pecan, Robusta, EPO, and Mugsy programs produced WGAs that were transitively closed and therefore unaffected by transformation. As predicted, those submissions that were not initially transitively closed, such as the pairwise and reference-based alignments, all saw their precision performance decline, in some cases very substantially, and no submission saw a large boost in recall.

### Missing duplications

To find duplications within the alignments, we used a simple metric, duplicative coverage. For a pair of genomes A and B, the duplicative coverage of B on A is the proportion of residues in A aligned to two or more distinct residues in B. This assessment is complicated by the lack of transitive closure in some submissions, because a single residue may align to two or more residues in a genome, but in separate columns of the file. To avoid this complexity, we assessed the submissions after computing the transitive closure (which also made the computational task significantly easier). To avoid misrepresenting submissions, we dropped submissions from the assessment for which the transitive closure adversely (>0.05 change) affected the F-score or pseudo F-score. Supplemental Figure S9 shows the results; in short, we find that only Cactus had significantly nonzero duplicative coverages, e.g., just over 3% of all fly genome bases were found to be duplicated, on average, when looking at any other genome.

### A code and data repository to reproduce the simulation results

To aid future assessment, we have created an easy to evaluate benchmarking pipeline (available at http://compbio.soe.ucsc.edu/alignathon/). Unfortunately the PSAR analysis involved using a compute cluster, making it expensive for outside groups to repeat this assessment. However, given a MAF file of one of the simulated data sets, the benchmarking pipeline can be used to make a performance assessment. The user can download the analysis repository, compile the necessary software, download the requisite data, place their alignment in a specified subdirectory and type “make” in the terminal window to launch the analysis. This approach will hopefully spur future development and assessment upon this resource.

## Discussion

With the explosion in sequencing delivering ever larger numbers of near complete genome assemblies, WGA is an essential and increasingly important task. We have tested a total of 35 submissions from 12 different pipelines across three different data sets to produce the largest and most comprehensive assessment of WGA to date. The assessment purposefully chose test genomes in the 100–200 Mb range. The decision to use data sets at this size scale, rather than at the scale of larger vertebrate genomes, was balanced, just as in the first Assemblathon, by the desire to attract the largest possible number of entrants while still creating a reasonable challenge.

The primate simulations indicated that for closely related genome sequences, aligners can find the vast majority of homologies accurately. In contrast, the simulated mammalian alignments showed a broader distribution of results. In concordance with this, we find that accuracies were substantially higher between more closely related genomes and higher in more conserved regions, even in alignments also involving more distantly related genomes—this was apparent both in looking at F-scores in the simulated mammals (Fig. 3) and pseudo F-scores in the flies (Supplemental Fig. S3). We also find via simulation that more highly conserved sequence is easier to align, and that duplications are poorly represented in current WGAs.

Testing using both simulations and real data, we find a clear concordance between the rankings. In addition, using the simulated data sets we were able to demonstrate reasonable linear correlations, both overall and regionally, between F-scores and pseudo F-scores. This indicates that the high-level aggregate differences we highlight between the submissions can be found by two entirely independent means. We did not find a linear correlation between precision and the statistical measure of precision we used (PSAR-precision, see below), but we did find a very strong correlation between recall and coverage. Importantly, for the submissions we received on both flies and simulated mammals, differences in recall were overall greater than differences in precision, and therefore more critical in determining the observed performance differences. We speculate, given the various overlaps in the tools used between the pipelines and the resulting similarities between the submissions, that the larger differences in recall were largely due to differences in synteny mapping, and that this is one area where there is clear room for improvement. Visualizing the data regionally, we were able to observe local differences in performance that fit well with these overall results.

For simplicity of interpretation in the simulations, homologies that predated the MRCA of the extant genomes were not included in the “true” simulated alignments; therefore, some ancient homologies captured by the aligners are considered false by the benchmarking pipeline. Additionally, EVOLVER does not track the alignments it generates when creating simulated mobile element (e.g., transposon) insertions; thus two highly similar transposon copies from separate insertion events are not considered homologous in the true alignment. For these reasons, the reported simulated precision values may be considered a lower bound that may, for some purposes (detecting ancient and mobile element-mediated alignments), underestimate the accuracy of the alignments. This may partly explain the lack of correlation between precision and PSAR-precision, because such repeat regions might appear to be reasonably alignable to PSAR, but false homologies according to the simulation.

There were some dependencies between the assessments and the assessed programs. EVOLVER simulations were used to benchmark the Cactus aligner in its initial publication, although at ∼1/250th the scale used here (Paten et al. 2011), and as part of two separate simulation assessments in that paper. It is therefore difficult to know if its substantial increase in relative performance is partially an artifact of training Cactus to the EVOLVER evolutionary model, although we note Cactus also performed well in the independent fly assessments. Similarly, PSAR uses the same pair-HMM alignment model as used by the PSAR-Align team in generating their alignments. We might expect therefore that the PSAR-Align alignments would be judged most accurate by PSAR, although we actually found a number of other programs earned equivalently high results.

The use of the MAF format for submissions made an apples-to-apples comparison somewhat difficult because the format does not force transitivity of homology. However, this permissiveness in format allowed us to assess a variety of WGAs, some of which are naturally not transitively closed, such as the reference-based MULTIZ aligners and the GenomeMatch pairwise submissions. To make comparisons under a strict homology assumption, we tried taking the transitive closure of such alignments, but this does not generally result in a reasonable WGA. In general, when performing consistent evolutionary analyses the nature of the alignment relationship—similarity or homology—bears consideration.

In theory, several of the tools should have been able to align duplicated regions together. Unfortunately, we received submissions only for the simulation and not fly data sets for some duplication-aware tools, such as EPO and Mugsy, but across the pipelines the lack of aligned duplications indicates there is likely significant room for WGA tools to improve in detection of duplicative homologies.

Some individual results of the simulations were surprising. For example, the EPO results had particularly low coverage on the simulations—substantially lower than that pipeline achieved in the genome alignments available from Ensembl (Paten et al. 2008). One possibility put forward by the investigators of EPO is that the tool’s reliance on using highly conserved sequences (“anchors”) for constructing a synteny map was not well suited to the simulations, which although modeled constraint, were likely different from the vertebrate genomes to which the EPO pipeline is normally applied.

## Conclusion

Robust WGA tools are critical for the future of comparative genomics, but to make objective progress we must agree on assessments or risk not knowing when a genuine advance has been made. As is typical in bioinformatics, much prior assessment of WGAs has been made as part of the publication of a novel tool. Naturally, these assessments tend to present results that favor the new tool. The few independent assessments that have been made of WGA, although useful, are several years old and assessed only a fraction of the available methods (Margulies et al. 2007; Chen and Tompa 2010). In comparison, the Alignathon has been a success; it has leveraged a collaborative-competition model that has had broad community involvement and led to a broader set of WGAs being compared than in any prior attempt and certainly more than any single research group would likely have had the patience or expertise to handle.

Just as in any area where there is no accepted ground truth, comparing WGAs is hard, and each of the assessment types categorized in the introduction has flaws. Here we put an emphasis on two independent methods for assessment and showed some consistency between them across data sets. For this reason, and because many of the results met with our prior expectation, we have some confidence in the results. Indeed, it is possible to compare the various F-scores and pseudo F-scores (Figs. 3–5, 7; Supplemental Figs. S1–S3, S7) and see some pipelines performed particularly strongly. However, given the uncertainty about the realism of the simulations and the apparent limited resolution of our statistical metrics, we caution against overinterpretation. Therefore, more independent lines of assessment need to be developed: more simulations, more statistical assessments, and more assessments at different scales (e.g., full mammalian genomes), etc.

Assessments like the Alignathon are useful to spur community activity. However, these kinds of benchmarking exercises risk becoming one-offs whose results are not comparable with the next generation of tools. To avoid this, we have tried to make the simulation assessments developed here easily reusable so that they might be included in future publications. There is then a risk that tools may become overfit to these benchmarks, therefore updating the benchmarks periodically is essential.

In the Supplemental Material, each of the teams describes how they computed each submission, which should be useful for reproducing their results. As the submissions were computed independently by each team and each team had a different hardware environment, we cannot fairly compare the computational cost of the different pipelines. It would be useful for future efforts to assess this aspect, perhaps by getting groups to run their aligners on a common platform, such as Amazon EC2 or Microsoft Azure, where a controlled comparison could be made. This may prove to be an optimistic goal though, because many WGA pipelines are designed and implemented at individual institutions by researchers whose goal is the sharing of the results of the pipeline but not the pipeline itself. The computation-environmental peculiarities of individual institutions can thereby be reflected in their pipelines through unintentional design.

We have left a number of questions unresolved. For example, we have not attempted to determine how tools for WGA compare to methods for other types of MSA, such as protein aligners, or how the quality of the input genome assemblies affects WGAs. In summary, we very much hope that the Alignathon will help pave the way for subsequent efforts with more data sets, comparisons, accurate statistical assessments, and benchmarking exercises with even broader scope.

## Methods

### Simulations

As in the Assemblathon 1 project (Earl et al. 2011), simulated genomes were generated using the EVOLVER suite of tools’ forward-time whole-genome evolution simulation tools (Edgar et al. 2009, http://www.drive5.com/evolver/). Specific parameter files used to create the simulations are available on the project website. EVOLVER has a model for proteins, genes, and base-level evolutionary constraints. EVOLVER uses a two-step process for simulating a single forward step in a simulation: The first step is an intrachromosomal evolution step, and the second is an interchromosomal step. The intrachromosomal step allows events such as substitutions, insertions and deletions, duplications, translocations, and inversions, according to rates distributed according to the length of the event. The interchromosomal step allows chromosome fusions, fissions, segment copying, segment movement, reciprocal translocations, and nonreciprocal translocations. Additionally, EVOLVER keeps a separate mobile element library that can insert mobile element DNA into the simulated genome; this library is itself also undergoing simulated evolution. EVOLVER logs all evolutionary events that take place during a cycle and keeps track of the relationships between residues in the parent and child genomes.

EVOLVER as distributed is only capable of performing a single cycle of evolution. In order to run the arbitrary phylogenies necessary for this project, we used the evolverSimControl and evolverInfileGeneration tools available at https://github.com/dentearl/evolverSimControl and https://github.com/dentearl/evolverInfileGeneration/, respectively. These extra tools, along with mafJoin (https://github.com/dentearl/mafJoin/) were used to construct MAF files containing the entire simulated true evolutionary relationships of all of the genomes: leaves, internal nodes, and the root.

As in the Assemblathon project, we initiated the simulation using a subset of the well-annotated human genome, hg19/GRCh37. Complete chromosome sequences for chromosomes 20, 21, and 22 along with annotations for those chromosomes from the UCSC Genome Browser tracks mgcGenes, knownGene, knownGeneOld5, cpgIslandExt, and ensGene, were obtained from the UCSC Golden Path download site. The tool suite evolverInfileGeneration was used to take the raw data and make it into an EVOLVER infile data set. This starting data set was then put through the EVOLVER simulator for a distance of 1.0 neutral substitutions per site, an evolutionary time of ∼500 million years of vertebrate evolution (Hedges et al. 2006; Earl et al. 2011; Fujita et al. 2011). This process, which we term a burn-in, shuffles the sequences, genes, and chromosomes of the genome. The resulting genome was termed the most recent common ancestor (MRCA), because it was used as the starting point for both the primate and mammalian simulations. It has been previously ascertained that distributions on the numbers and lengths of tracked annotation types in EVOLVER simulations stay stationary over time (Earl et al. 2011), so this burn-in process, from a simulation point of view, does not adversely affect the nature of the simulated genomes.

EVOLVER rediscovers the tandem repeat sequence annotation at every step of a simulation by calling tandem repeats finder (Benson 1999, v4.0). RepeatMasker (Smit and Hubley 2010, v1.25; Smit et al. 2010) and tandem repeats finder were used to identify and soft-mask repetitive sequence in the final leaf genomes.

The primate simulation was described by the phylogenetic tree (in newick format) (Fig. 1): ((simGorilla:0.008825,(simHuman:0.0067,simChimp:0.006667)sHuman-sChimp:0.00225)sG-sH-sC:0.00968,simOrang:0.018318). The mammal simulation was described by the phylogenetic tree (in newick format) (Fig. 1): ((simCow:0.18908,simDog:0.16303)sCow-sDog:0.032898,(simHuman:0.144018,(simMouse:0.084509,simRat:0.091589)sMouse-sRat:0.271974)sH-sM-sR:0.020593).

We used the EVOLVER produced repetitive element library from the simHuman genome as an input library for RepeatMasker. Following each simulation, the EVOLVER mobile element library from the simHuman leaf node genome was used as an input into the repetitive sequence finder RepeatMasker. RepeatMasker was then used to mask simple repeats and repeats from the provided library in the other nonhuman simulated genomes.

Complete sequence and annotations of the leaf genomes and the MRCA genome were provided to participants.

### Fly data set

The phylogeny was created by merging the phylogeny provided in the modENCODE (The modENCODE Consortium et al. 2010) comparative genomics white paper (http://www.genome.gov/Pages/Research/Sequencing/SeqProposals/modENCODE_ComparativeGenomics_WhitePaper.pdf; accessed October 15, 2013) courtesy of Artyom Kopp (UC Davis) and the phylogeny used by UCSC for the 15-way insect alignment. The Kopp tree lacked droSim1 and droSec1 which were added by normalizing the branch lengths between the dm3 branches on the two trees. Extraneous species were trimmed using tree_doctor from PHAST. This tree was provided for progressive aligners that need a guide tree. This phylogeny corresponds to the newick tree (Fig. 1): ((droGri2:0.183954,droVir3:0.093575):0.000000,(droMoj3:0.110563,((((droBip:0.034265,droAna3:0.042476):0.121927,(droKik:0.097564,((droFic:0.109823,(((dm3:0.023047,(droSim1:0.015485,droSec1:0.015184):0.013850):0.016088,(droYak2:0.026909,droEre2:0.029818):0.008929):0.047596,(droEug:0.102473,(droBia:0.069103,droTak:0.060723):0.015855):0.005098):0.010453):0.008044,(droEle:0.062413,droRho:0.051516):0.015405):0.046129):0.018695):0.078585,(droPer1:0.007065,dp4:0.005900):0.185269):0.068212,droWil1:0.259408):0.097093):0.035250).

To create the fly sequence data set, we took 12 flies available from the UCSC golden path server on 14 December 2011 (droAna3, dreEre2, droGri2, droMoj3, dp4, droVir3, droWil1, dm3, droSim1, droPer1, droSec1, droYak2) and eight flies from NCBI on January 25, 2012 (droBia, droBip, droEle, droFic, droKik, droTak, droRho, droEug).

### MafTools

Participants submitted their predictions of alignments in MAF files. To process the submissions, we wrote a suite of open-source tools called mafTools (available at https://github.com/dentearl/mafTools/) to perform the majority of transformations, manipulations, and analyses. Scripts to perform the analyses described can be found in the analysis repository.

### MAF comparisons

Exhaustively checking all pairs of aligned residues between alignments is computationally impractical, so instead we developed a method, termed *mafComparator*, based upon sampling pairs of aligned residues. Sampling is performed by reading each input MAF file twice, once to count the total number of pairs present in the file, such that given a user specified number of pairs to sample we can calculate the probability of picking a given pair at random. The MAF file is then read a second time.

During the second pass we iterate over every block in the MAF and then every column in the block. We calculate the number of pairs present in the block, call this *k*, and then make a draw from a binomial distribution with probability *s*/*m* (where *s* is the number of samples taken, here 10,000,000, and *m* is the total number of pairs present in the MAF) to see how many (if any) pairs to sample from that column. If *x* many pairs are to be sampled, we then sample *x* times from a discrete uniform [0, *k* − 1] decrementing the range of the distribution with each sample, without replacement, and then map those integers to pairs using a bijective function. This allows us to efficiently sample pairs without iterating through each and every pair.

### Regional alignments and PSAR

To accommodate PSAR, which processes global MSAs in which the alignment is represented as a 2D matrix where the aligned sequences, interspersed with gaps, are the rows and the columns represent the equivalence classes of aligned bases, we constructed subalignments of sampled regions.

Regions were randomly sampled by picking intervals of a chosen reference genome (for the flies *D. melanogaster,* dm3, and for the simulations, simHuman). For each of the three test sets, regional intervals were selected by sampling five different starting values from a discrete uniform distribution (0, *g* − 1 − 500,000), where *g* is the total length of the reference genome and 500,000 is the length of the interval. Sampled values were then mapped back to individual chromosomes. All alignments containing any positions of the reference within these intervals were extracted from the submitted alignments. Although this model of sampling does not prevent overlapping regions, no overlapping regions were sampled. Likewise this model of sampling does not prevent regions that cross between chromosomes, but no such bridged regions were sampled.

Details of how we adjusted each submission for regional analysis with PSAR are in the Supplemental Material.

## Data access

The project website is available at http://compbio.soe.ucsc.edu/alignathon/. This website links to all the data sets, submissions, and benchmarking code.

## Supplementary Material

Supplemental Material
