# Supplementary material for: Alignathon: a competitive assessment of whole-genome alignment methods
Source: Genome Res. 2014 Dec;24(12):2077–89. doi: 10.1101/gr.174920.114 (PMC4248324; doi:10.1101/gr.174920.114)
Supplement: Supplemental Material [file supp_24_12_2077__index.html]

Alignathon: a competitive assessment of whole-genome alignment methods — Alignathon: a competitive assessment of whole-genome alignment methods — Supplemental Material 

# Alignathon: a competitive assessment of whole-genome alignment methods

## Supplemental Material

**Files in this Data Supplement:**

- Supplemental Material and Figures.pdf
