# Supplementary material for: Alignathon: a competitive assessment of whole-genome alignment methods
Source: Genome Res. 2014 Dec;24(12):2077–89. doi: 10.1101/gr.174920.114 (PMC4248324; doi:10.1101/gr.174920.114)
Supplement: Supplemental Material [file supp_gr.174920.114_Supplemental_Material.pdf]

Alignathon Supplement

The Alignathon Authors

September 25, 2014

Contents

0.1 Details of Regional Alignments & PSAR 4
0.2 Details of Submissions 5
    0.2.1 AutoMZ and TBA 5
    0.2.2 Cactus 7
    0.2.3 EPO (Enredo Pecan Ortheus), mammals simulation . 7
    0.2.4 Pecan (Mercator-Pecan), primates simulation 8
    0.2.5 Pecan, mammals simulation 8
    0.2.6 GenomeMatch 9
    0.2.7 Mugsy 10
    0.2.8 MULTIZ primates 10
    0.2.9 MULTIZ mammals 10
    0.2.10 MULTIZ flies 11
    0.2.11 progressiveMauve 11
    0.2.12 PSAR-Align 11
    0.2.13 Robusta 12
    0.2.14 VISTA-LAGAN 14
0.3 Supplemental Figures and Tables 14

List of Figures

S1 Region 1 of simHuman regional analysis of the sim. primate data set. 22
S2 Region 4 of simHuman regional analysis of the sim. mammal data set. 23
S3 Pseudo F-score stratified by phylogenetic distance for fly data set. 24
S4 Correlation between PSAR-based pseudo-F-Scores and F-Scores. 25
S5 Correlation between coverage and recall. 27
S6 Correlation between PSAR precision and precision. 28
S7 Pseudo F-score for the regional analysis of sim. mammal data set. 29
S8 Change in F-Score between original submissions and transitive closure 30
S9 Overall average prop. of duplications for transitively closed submissions. 31
S10 Overall average prop. of duplications for original submissions. 32
S11 Correlation of values between regional and whole genome analyses. 36

List of Tables

S0 AutoMZ and TBA submission runtime details 6
S1 A listing of simulated genome events for the two phylogenies. 15
S2 Summary statistics of submissions for the primate data set. . 16
S3 Summary statistics of submissions for the mammal data set. . 16
S4 Summary statistics of submissions for the fly data set. 17
S5 Primate simulation F-score results. 18
S6 Mammal simulation F-score results. 19
S7 Mammal simulation F-score results stratified by annotation type. 20
S8 Primate simulation F-score results stratified by annotation type 21
S9 Fly pseudo F-score regional results. 26
S10 Jaccard distance values for all submissions to the primate dataset. 33
S11 Jaccard distance values for all submissions to the mammal dataset. 34
S12 Jaccard distance values for all submissions to the fly dataset. 35
S13 Primate simulation precision results. 37
S14 Primate simulation recall results. 38
S15 Mammal simulation precision results. 39
S16 Mammal simulation recall results. 40
S17 Mammal simulation precision results stratified by annotation type. 41
S18 Mammal simulation recall results stratified by annotation type. 41
S19 Primate simulation precision results stratified by annotation type. 42
S20 Primate simulation recall results stratified by annotation type. 42

0.1 Details of Regional Alignments & PSAR

To convert each subregion into a 2D alignment matrix we designed a pipeline that used the chosen reference genome interval to create a "reference alignment" as follows:

1. For each submission, for each region, we used `mafExtractor` to pull out maf blocks that contained any positions in the reference that were within the target region. These blocks were then trimmed to contain only positions that aligned to the reference within the region of interest.

2. We used the `mafTransitiveClosure` tool to compute the transitive closure of the resulting alignment (see discussion), to make each alignment a consistent multiple sequence alignment. This was particularly important for the GenomeMatch alignments, which were all pairwise. Unlike for the whole genome alignments, taking the transitive closure of the aligned pairs just within the subregion did generally not perturb the performance substantially (data not shown).

3. Next, a row deduplication step was performed using `mafDuplicateFilter`, so that each species contributed at most one row to the 2D alignment. In short, for every block in the alignment a consensus sequence is created and then for species with multiple instances present in the block a similarity score is calculated in relation to the consensus. Only the sequence closest to the consensus in terms of substitution distance is kept. In the event of a tie the sequence closest to the start of the block is used. All the other instances are discarded.

4. The previous step could result in the removal of the reference sequence that tied the block into the region of interest. As a result, a second step of reference oriented region based extraction was performed to eliminate all blocks that more strongly align to an area outside of the region of interest (i.e. a part of the reference outside the region of interest had a higher similarity score in the row deduplication step).

5. The blocks were sorted based upon their left-right order along the positive strand of the region of interest in the reference. Then the rows of all blocks were reordered to be standardized (into a consistent species order, i.e. alphabetically) and finally all of the reference sequence instances were forced to be in relation to the positive strand.

Rearrangements in the non-reference sequences were ignored by concatenating the fragments of the non-reference sequence together according to their ordering along the reference sequence, as is standard practice in constructing "reference" alignments.

To ascertain how these manipulations affected the alignments we calculated regional precision and recall values for the simulated subregions, as we did for the larger alignments. Looking at the mammals, we find reasonable linear correlations between regional and overall precision ($r^2 = 0.594$), recall ($r^2 = 0.989$) and F-score values ($r^2 = 0.992$) (Supplemental Figure S9), suggesting that the alignment manipulations did not bias the results too substantially. Furthermore, we find for most submissions a low variance in these results between different regions, suggesting five regions were likely enough to get a good approximation of the overall results.

To run PSAR on the regional alignments we broke up each regional alignment into 2kb windows and ran PSAR independently on each window. This windowing is likely to have somewhat affected the scores, though the effect was likely a very minor effect as the number of breaks between chunks is very small compared to the number of columns in the alignment. To make the scores comparable between different alignments, we look only at pairs including the reference sequence, which is a shared constant —and thus ignored non-reference pairs.

0.2 Details of Submissions

Each group was asked to detail how they computed their submissions, the computational resources used and the runtimes involved. What follows are the responses of the participating groups.

0.2.1 AutoMZ and TBA

I used LASTZ to produce pairwise alignments. AutoMZ and TBA are slightly modified from the package available at `http://www.bx.psu.edu/miler_lab`, with no major algorithmic change. AutoMZ is reference-dependent and progressively aligns species in the species tree using the reference sequence as the guide. TBA progressively aligns species in the tree without a specified reference.

The overall steps of the pipelines are: running lastz to get pairwise alignments, then use single_cov2 (in the MULTIZ/TBA software package) to post-process such pairwise alignments, and finally run AutoMZ or TBA

	Primates	Mammals	Flies
AutoMZ	90 minutes	220 minutes	27 hours
TBA	125 minutes	204 minutes	7 days*

Table S0: AutoMZ and TBA submission runtime details. *The program got very low priority on the server and other computation-extensive programs ran on the same sever for unknown time.

to produce multi-alignment. Among all steps, the first step to produce lastz alignment was most non-trivial in my pipeline. There were a few sequences (in flies) not repeat masked well, and lastz failed on the computer cluster I used. I finally got help from Bob Harris (at Penn State) to separately repeat mask these sequences in order to run lastz. Once lastz alignments were produced, the rest of steps were simply invoking automatic commands from the package.

For pairwise alignments, I used the following resource to set up the parameters of running LASTZ: `http://genomewiki.ucsc.edu/index.php/Hg19_conservation_lastz_parameters`. For species pairs not listed in the resource, I used default parameters.

For all multi-alignments, I used default parameters. For AutoMZ alignments, I used references suggested by the organizers [simHuman for both simulations, dm3 for flies].

The pairwise alignments were distributed on a computer cluster. The total amount of time would be at least thousands of hours. The time for multi-alignments was estimated based on the timestamps of files on the server. The server might have other programs running at the same time when computing these alignments.

Table S0 shows the run times of different programs used on the three different datasets.

Peak memory usage was not recorded. Most pairwise alignments were done on a computer cluster where each computer node has 1G memory. For the pairs of species that exceeded the memory, I splitted the sequences into smaller fragments and combined separate alignments later. The job requiring the maximum memory was producing TBA alignment of flies, which was done on a machine with 10G memory. So the peak memory use of the pipeline must be within 10G.

Pairwise alignments were computed on a computer cluster of around 100 processors, each with 1G memory. Multi-alignments were computed on a server with configured maximum 10G memory.

Pairwise alignments used a batch system. Multi-alignments used a single machine.

One person spent approximately 100 person-hours spent on these submissions. Most of time was spent on obtaining the pairwise alignments, since some of sequences were not repeat masked well, the pipeline broke many times when running on the computer cluster. It took time to track and fix them. Once the pairwise alignments were computed, it didn't take too much effort to get the multi-alignment results.

0.2.2 Cactus

All three datasets were aligned using the default parameters of progressive-Cactus. Progressive cactus differs from the original Cactus program (described in (Paten, Earl, Nguyen, Diekhans, Zerbino & Haussler 2011)) because it uses a progressive alignment strategy to align the genomes using a guide tree, employing the Cactus alignment algorithm at each step. In each case the guide tree used was the one provided (shown in Figure 1). Progressive cactus can be found at: `https://github.com/glennhickey/progressiveCactus/`. The alignments were computed on a 64 core machine with 1 terabyte of ram. Only CPU hours were recorded for the simulated mammal dataset - and totalled just over 500 hours total.

0.2.3 EPO (Enredo Pecan Ortheus), mammals simulation

EPO stands for Enredo-Pecan-Ortheus. Enredo uses a set of anchors mapped on the genomes. For a given set of species, we have to generate a specific set of anchors. Note that you can re-use the same set for different runs, if you want to add a new sequence for instance. The anchors are extracted from conserved regions identified in sets of pairwise alignments. GERP is used to find the most conserved region in long alignments. All anchors are mapped on every genome. Hits are filtered and sent to Enredo. Enredo is a graph-based method that will define blocks of collinear sequences. These blocks can contain segmental duplications. Collinear segments are aligned with Pecan/Ortheus. Ortheus relies on the species tree when no duplication is detected in a given block. For the other blocks, it uses a recursive loop to find the best guide tree/alignment, starting with a random guide tree and using Semphy to infer a tree from the alignment.

Pecan was run as Ortheus. We use Ortheus to infer ancestral sequences. Ortheus runs Pecan internally. Ortheus was run with default parameters.

124.0 CPU hours (pairwise alignments)

744.6 CPU hours (anchor generation)a
220.8 CPU hours (anchor mapping)
$<$ 0.1 CPU hours (Enredo)
58.3 CPU hours (Pecan/Ortheus)
Wall-clock time was 2:20 hours (Pecan/Ortheus only)
Peak memory was 3397 Mb (Pecan/Ortheus only)
The default Sanger compute farm was used:
Machines Cores per machine CPU type Memory
1404 2x2.0 Ghz dual core Opteron 2708GB
64 8 2x1.8 Ghz quad core Intel L5320 16GB
56 4 2x2.6 Ghz dual core Intel 5150 8GB
238 8 2x3.0 Ghz quad core Intel E5450 16GB
128 12 2x 2.6 Ghz hex core Intel X5650 36GB
168 12 2x 2.6 Ghz hex core Intel X5650 36GB
We use eHive to manage the jobs. Processes in the farm are controlled by LSF.

One person worked on this submission.

Enredo's parameters have been optimised for real mammalian genomes. We have noticed that the coverage on the simulated genomes is much lower than with real genomes.

0.2.4 Pecan (Mercator-Pecan), primates simulation

Sequence similarities among coding genes are used to build an orthology map with Mercator. Collinear segments are aligned with Pecan.

Mercator was run by Carsten Kemena from Cédric Notredame's group (CRG).

Pecan was run with default parameters.

Pecan used 14.2 CPU hours, 3:05 wall-clock hours.

The Sanger default compute farm was used (see above for specs)

We use eHive to manage the jobs. Processes in the farm are controlled by LSF.

Two people worked in this submission. We wish to thank Carsten Kemena for providing Mercator results.

0.2.5 Pecan, mammals simulation

Sequence similarities among coding genes are used to build an orthology map with Mercator. Collinear segments are aligned with Pecan.

Mercator was run by Carsten Kemena from Cédric Notredame's group (CRG).

Pecan was run with default parameters.

Pecan used 49.5 CPU hours, 0:36 wall-clock hours, 2640 Mb of memory (peak).

Sanger default compute farm was used (see above for specs).

We use eHive to manage the jobs. Processes in the farm are controlled by LSF.

Two people worked in this submission. We wish to thank Carsten Kemena for providing Mercator results.

0.2.6 GenomeMatch

The GenomeMatch pipeline is developed to find alignments between genome sequences as well as to construct synteny maps. At the initial stage it generates a list of all homology blocks with length > 35 and similarity $> 70\%$. As preliminary local alignments we consider consecutive chains of blocks that closer 20000 and length of gaps < 200000. On the next stage we identify alignments of smaller blocks within intervals between blocks found on the initial stage and add them to preliminary alignments forming a set of final local alignments.

To build synteny we apply dynamic programming to final local alignments to find the best set of non-overlapping alignments.

We computed alignments applying default parameters that were initially optimized using simulated similar sequences.

The alignment of 4th chromosome of mouse with 1st chromosome takes ˜35 minutes of one core of 2.3 GHz Intel processor. The GenomeMatch runs alignment of different sequence pairs on different cores of a multi-processor computer. Each sequence pair requires ˜0.3 - 3 GB of computer shared memory. For analysis of the competition data we used single shared memory machine with 12 dual-core processors and 256 GB memory. The GenomeMatch loads alignment of different sequence pairs to different cores of the multi-processor computer.

Approximately 40 person-hours were spent on these submissions.

Alignment of 'dm3.fa' (165 Mb, 15 sequences) and 'dp4.fa' (149 Mb, 4896 contigs) from 'packageFlies' takes ˜18 minutes using ˜5.6 Gb (˜700 Mb per process) of physical memory and 8 processor cores of Intel Xeon CPU X5690 @ 3.47GHz.

Alignment of 'simHuman.fa' (181 Mb, 4 chromosomes) and 'simChimp.fa' (181 Mb, 4 chromosomes) from 'packagePrimates' takes ˜2 hours using ˜9.6

Gb (~2.4 Gb per process) of physical memory and 4 processor cores of Intel Xeon CPU X5690 @ 3.47GHz.

0.2.7 Mugsy

Mugsy x86-64-v1r2.2 was used in single threaded mode with entirely default parameters for both the simulated datasets. Both datasets took approximately one day of compute time. Though parameter adjustment may have been able to improve the sensitivity somewhat the submitter is not convinced that MUMmer would have been able to adequately deal with the degree of divergence in the simulated mammals dataset. The default maximum unique match (MUM) size in the version used was 15, which appears to be quite low. It is the submitter's opinion that there is not much room to improve sensitivity without moving to approximate string matching.

0.2.8 MULTIZ primates

The standard UCSC MULTIZ pipeline was used. The resulting alignment is reference based. The first step is to build pairwise alignments using lastz which are chained using axtChain, then further filtered using chainNet. The resulting chains are then given to MULTIZ which generates the multiple alignment. (Kent, Baertsch, Hinrichs, Miller & Haussler 2003, Blanchette, Kent, Riemer, Elnitski, Smit, Roskin, Baertsch, Rosenbloom, Clawson, Green et al. 2004)

Default parameters to lastz.

Minimum chain score: 3000 (slightly conservative)

Chain linear gap costs: medium (default)

Net used: standard net

Chaining 1435 CPU hours, 8 wall clock hours, 4 GB peak memory.

MULTIZ 1 hour, 1 hour, 8 GB peak memory.

The UCSC swarm cluster was used, utilizing 512 cores, 4 GB memory per 2 cores, running the parasol job batching system. One person worked on this submission for a total of five hours.

0.2.9 MULTIZ mammals

Chaining took 455 CPU hours, 3 wall clock hours, 4 GB peak memory.

MULTIZ took 2 CPU hours, 1 wall clock hour, 8 GB peak memory.

0.2.10 MULTIZ flies

Chaining took 903 CPU hours, 33 wall clock hours, 4 GB peak memory.

Mutliz took 14 CPU hours, 4 wall clock hours, 8 GB peak memory.

0.2.11 progressiveMauve

The progressiveMauve multiple genome alignment software calculates positional homology alignments among two or more DNA sequences (Darling, Mau & Perna 2010). The algorithm does not align paralogs to each other, but does align the positionally-conserved copy of repeat sequences in different genomes.

The progressiveMauve software is a single standalone program for 64-bit Linux that incorporates the following steps: (1) identify unique multi-matches among all input sequences, (2) construct local chains of matches called Locally Collinear Blocks, (3) multiple-align Locally Collinear Blocks, (4) filter out low quality alignments with a homology HMM. Much more detail is available in the publication:

Default parameters were used. The software version is the February 2nd 2011 build. (e.g., predating alignathon by 1 year).

4.45 CPU hours, 4.46 wall-clock hours were used. Peak memory was 11.2GB.

1 CPU on a shared memory machine was used.

One person spent three person-hours for alignment, four person-hours for writing an XMFA to MAF converter.

Starting from the primate sequences directory, the command used to generate the alignment is:

`progressiveMauve --output=primate *.fa`

Timing statistics were collected with `/usr/bin/time -v`

Time to convert from XMFA to MAF was not included but is negligible, a few minutes at most.

Results for the mammals simulation were nearly complete when a power failure interrupted the program and all results were lost. The program had been running for several weeks and used at least 200GB of memory. It's fair to say this implementation doesn't scale reasonably to such datasets.

0.2.12 PSAR-Align

For each MAF alignment fragment in given a MAF alignment, the Gblocks program was used to find highly conserved anchors with length $\geq$ 200bp by using default options of the Gblocks program. Then, for each inter-anchor

regions, (i) PSAR was run to sample sub-optimal alignments, (ii) posterior probabilities of aligning two residues from two different sequences were computed, and (iii) finally revised alignments were built from the posterior probabilities.

Both simulated datasets were run on a compute cluster, utilizing 200 Intel(R) Xeon CPU 2.67GHz cores with 1GB memory for each job.

Primates: 13 hours wall clock.

Mammals: 119 hours wall clock.

0.2.13 Robusta

We used the Mercator program to split the genomes into blocks. As input we used the provided gff files for the simulated sets and predictions from Geneid as well as data from UCSC for the drosophila sets. The blocks were then aligned using the Robusta alignment program (not published). Robusta is a meta-aligner and an extension of the M-Coffee package that combines the output of several alternative aligners into one unique final model.

Alignment:

Drosophila:

We used the Robusta program we developed to calculate different combinations of existing alignment methods (Pecan, Mavid, progressiveMauve, Lastz). For this the different methods were called pairwise on the different datasets and the resulting alignment combined using the T-Coffee consistency algorithm.

In order to identify the best combination of methods, we used available RNA-Seq data (Odorant receptor, larval stage) collected on 6 of the 20 considered species (see below). Mapped read patterns were then projected onto the sequences and their matches were used to define an objective function. This function was used to compute the score of every block containing enough mapped RNA-Seq data. For this analysis we used two alternative protocols for the selection of the alignments:

1)AverageBest

All blocks were produced with the same method which gave us the best average score and the best scoring combination of primary methods was selected. In our test this was Pecan-Mavid-Lastz which combines the three the considered packages,

2)PickBest

For this method we picked for each block the method which gave the best RNA-Seq score. Whenever not enough RNA-Seq data was available,

we picked up the AverageBest combination. The following alignment methods were considered: Robusta: Pecan- Mavid-Lastz, Pro-Coffee, Pecan, Robusta: Pecan-Lastz, Robusta:Pecan-Mavid- pMauve, Robusta: Lastz, Robusta: Pecan-Mavid, Robusta: Mavid-Lastz, Robusta: Pecan, Robusta: pMauve-Lastz, Robusta: Pecan-pMauve-Lastz, Robusta: Mavid, Robusta: Pecan-Mavid-pMauve-Lastz, Mavid, Robusta:Pecan-pMauve, Robusta:pMauve, Robusta: Mavid-pMauve, Robusta: Mavid-pMauve-Lastz

Simulated:

We used the method which on average gave the best RNA-seq value for the fly set (Robusta-Pecan-Mavid-Lastz).

Alignment (simulated sets)

We used the method which on average gave the best value for the fly set (Rpw-Pecan-Mavid-Lastz).

Evaluation using RNA-Seq

We have RNA-Seq data for 6 of the 20 fly species (dp4, droAna3, droEre2, droWil1, droYak2, droVir3). We mapped the reads using the segemehl program with default settings. We limited the measurement of accuracy to those sites in the data where a strong difference in the number of mapped reads (either drop or increase) occurs. We then scored each pair of aligned nucleotides with 1 when both nucleotides show an increase/decrease or with -1 in case of matching an increase with a decrease. For each pair of sequences the values are summed up to produce the final score of this alignment.

CPU Hours

Primate:1d 8h 44m

Mammal: 3d 13h 12m

Fly:

AverageBest: 52d 10h 25m

PickBest: 518d 3h 10m

Wall-clock time was not recorded.

Peak memory was not recorded, but the maximum memory available at any time was 150 Gb

Nine machines, having between 4 and 12 cpus, between 4 and 150GB Memory were used.

The genomes were split and the resulting blocks were then run on single machines.

Five people worked on these submissions.

0.2.14 VISTA-LAGAN

We aligned three datasets: simulated Test set, simulated Primate set, simulated Mammal set.

VISTA pipeline infrastructure (Frazer, Pachter, Poliakov, Rubin & Dubchak 2004, Dubchak, Poliakov, Kislyuk & Brudno 2009) was utilized for the construction of genome-wide multiple DNA alignments between all genome assemblies. VISTA uses an efficient combination of global and local alignment methods and consists of the following steps: obtaining a map of large blocks of conserved synteny between the two species by applying Shuffle-LAGAN glocal chaining algorithm (Brudno, Malde, Poliakov, Do, Couronne, Dubchak & Batzoglou 2003) to local alignments by translated BLAT (Kent 2002); using Supermap, the fully symmetric whole-genome extension to the Shuffle-LAGAN. Alignment is done by PROLAGAN, a variation of the original Multi-LAGAN program that allows for the alignment of two alignments (profiles) and predicting ancestral contigs using a maximum matching algorithm (Dubchak et al. 2009). The four stages (local hits, chaining, global alignment, and ancestral reconstruction) are repeated for every node in the phylogenetic tree.

0.3 Supplemental Figures and Tables

Simulation	Substitutions	Deletions	Inversions	Moves	Copy	Tandem	Chr Split	Chr Fuse	Create CDS	Delete CDS	Create UTR	Delete UTR
Burnin	138967945	9658227	43101	16429	57467	4498368	6	4	42	36	32	29
Primates	3021621	213826	944	354	1269	98307	0	1	0	1	0	0
Mammals	27098495	1918761	9013	3653	11465	883944	2	1	5	4	2	5

Table S1: A listing of simulated genome events for the two phylogenies. Burnin represents the sum total of events from the starting input genome until the MRCA genome, a phylogenetic distance of 1.0 neutral subs per site. Primates represents the number of events between the MRCA and the simHuman genome, a phylogenetic distance of 0.01863 neutral substitutions per site. Mammals represents the number of events between the MRCA and the simHuman genome, a phylogenetic distance of 0.164611.

Submission	Sequence Chars (10^6)	Gap Chars (10^6)	Ave Block Area (10^3)	Ave # Rows in Blocks
AutoMZ	738 (98.1%)	14 (1.9%)	13.2	3.69
Cactus	1,100 (65.6%)	577 (34.4%)	2.17	7.58
GenomeMatch V1	2,000 (100%)	0 (0%)	0.752	2.00
GenomeMatch V2	2,000 (100%)	0 (0%)	0.713	2.00
GenomeMatch V3	8,500 (100%)	0 (0%)	0.198	2.00
Mugsy	740 (96.4%)	28 (3.6%)	33.6	2.25
MULTIZ	737 (98%)	15 (1.9%)	13.7	3.82
Pecan	740 (88.7%)	95 (11.3%)	3,450	4.00
PSAR-Align	735 (98.2%)	14 (1.8%)	11.9	3.64
progressiveMauve	741 (94.4%)	44 (5.6%)	519	2.67
Robusta	741 (90.8%)	75 (9.2%)	12,000	4.00
TBA	744 (98.1%)	14 (1.8%)	11.9	3.00
VISTA-LAGAN	776 (95.6%)	35 (4.4%)	9.71	3.77

Table S2: Summary statistics of submissions for the primate data set. Columns shown are: the number of sequence characters in megabases and parenthetically the percent relative to gap and sequence characters; the number of gap characters in megabases and parenthetically the percent relative to gap and sequence characters; the average block area where block area for a given block is defined as the product of the number of columns in the block and the number of sequences (rows) in the block; the average number of rows in blocks. Note that the GenomeMatch tool is a pairwise alignment tool and as such produces blocks containing exactly two sequences (the reference and the partner). Likewise the EBI-MP and Robusta submissions ensure that each block contains each of the four leaf genomes.

Submission	Sequence Chars (10^6)	Gap Chars (10^6)	Ave Block Area (10^3)	Ave # Rows in Blocks
AutoMZ	904 (73.3%)	330 (26.7%)	0.630	4.35
Cactus	1,380 (28.4%)	3,470 (71.6%)	1.04	7.36
EPO	826 (52.0%)	726 (48.0%)	545	5.17
GenomeMatch V1	359 (100%)	0 (0%)	0.168	2.00
GenomeMatch V2	631 (100%)	0 (0%)	0.169	2.00
GenomeMatch V3	375 (100%)	0 (0%)	0.170	2.00
Mugsy	685 (86.3%)	109 (13.7%)	5.00	1.4
MULTIZ	804 (77.8%)	230 (22.2%)	1.32	4.09
Pecan	948 (35.2%)	1,750 (64.8%)	3,520	4.84
PSAR-Align	797 (77.9%)	226 (22.1%)	0.986	3.87
Robusta	957 (37%)	1,630 (63%)	7,170	4.98
TBA	971 (74.8%)	328 (25.2%)	0.734	2.74
VISTA-LAGAN	991 (61.4%)	624 (38.6%)	4.72	4.21

Table S3: Summary statistics of submissions for the mammal data set. Columns are the same as in Supplemental Table S2. Note that GenomeMatch is a pairwise alignment tool and always contains exactly two rows in each block.

Submission	Sequence Chars (10^6)	Gap Chars (10^6)	Ave Block Area (10^3)	Ave # Rows in Blocks
AutoMZ	2,030 (54%)	1,730 (46%)	4,120	13.23
Cactus	2,170 (15%)	12,293 (85%)	1.02	12.50
GenomeMatch V1	14,300 (100%)	0 (0)%	0.194	2.00
GenomeMatch V2	17,900 (100%)	0 (0)%	0.211	2.00
GenomeMatch V3	31,800 (100%)	0 (0)%	0.244	2.00
MULTIZ	1,800 (57.4%)	1,340 (42.6%)	2.680	14.90
Robusta-AveBest	2,410 (19.7%)	9,850 (80.3%)	2,150	16.41
Robusta-BestPick	2,410 (20.1%)	9,570 (79.9%)	2,120	16.41
TBA	3,440 (66%)	1,800 (34.38%)	0.481	4.97

Table S4: Summary statistics of submissions for the fly data set. Columns are the same as in Supplemental Table S2. Note that GenomeMatch is a pairwise alignment tool and always contains exactly two rows in each block.

Name	F-score Overall	F-score Region mean	F-score Region std	Pseudo F-score Region mean	Pseudo F-score Region std
PSAR-Align	0.990	0.990	2.05e-03	0.988	1.21e-04
Mugsy	0.989	0.990	2.39e-03	0.987	3.42e-04
TBA	0.989	0.990	1.62e-03	0.990	7.96e-05
MULTIZ	0.988	0.989	2.13e-03	0.989	1.02e-04
AutoMZ	0.988	0.990	1.61e-03	0.989	7.90e-05
Cactus	0.987	0.987	1.74e-03	0.989	3.55e-05
progressiveMauve	0.986	0.987	6.59e-04	0.983	2.21e-04
VISTA-LAGAN	0.983	0.985	3.03e-03	0.988	1.43e-04
Pecan	0.969	0.983	8.18e-03	0.966	2.12e-03
Robusta	0.965	0.980	1.10e-02	0.971	3.34e-03
GenomeMatch-1	0.947	0.949	3.40e-03	0.944	1.61e-04
GenomeMatch-2	0.936	0.946	3.79e-03	0.945	2.81e-04
GenomeMatch-3	0.406	0.927	1.22e-03	0.946	3.43e-04

Table S5: Primate simulation F-score results. Rows are ordered by descending value of overall (full genome) F-score. Columns are: F-score overall value; F-score region mean; F-score region standard deviation; pseudo F-score region mean; pseudo F-score region standard deviation.

Name	F-score Overall	F-score Region mean	F-score Region std	Pseudo F-score Region mean	Pseudo F-score Region std
Cactus	0.803	0.787	1.17e-02	0.801	5.96e-04
PSAR-Align	0.722	0.729	1.14e-02	0.770	5.52e-04
MULTIZ	0.722	0.728	1.14e-02	0.774	5.08e-04
TBA	0.716	0.702	1.07e-02	0.826	5.01e-04
VISTA-LAGAN	0.712	0.696	7.91e-03	0.790	4.58e-04
AutoMZ	0.663	0.671	1.56e-02	0.819	1.12e-03
Pecan	0.536	0.546	1.68e-01	0.509	2.68e-02
Robusta	0.497	0.51	1.59e-01	0.553	2.84e-02
EPO	0.372	0.31	1.35e-01	0.351	4.93e-03
GenomeMatch-3	0.197	0.07	2.66e-02	0.167	3.39e-04
GenomeMatch-1	0.196	0.071	2.66e-02	0.165	2.88e-04
GenomeMatch-2	0.196	0.07	2.66e-02	0.166	3.23e-04
Mugsy	0.127	0.009	8.00e-03	0.112	2.15e-04

Table S6: Mammal simulation F-score results. Rows are ordered by descending value of overall (full genome) F-score. Columns are: F-score overall value; F-score region mean; F-score region standard deviation; pseudo F-score region mean; pseudo F-score region standard deviation.

Name	Overall F-score	Genic F-score	Neutral F-score	Repeats F-score
Cactus	0.7245	0.86781	0.73074	0.66848
PSAR-Align	0.66141	0.8532	0.67322	0.36298
MULTIZ	0.65806	0.8531	0.66992	0.35914
VISTA-LAGAN	0.65689	0.85415	0.66768	0.55966
TBA	0.65213	0.85399	0.66855	0.3872
AutoMZ	0.59437	0.84868	0.61423	0.20636
Pecan	0.59045	0.85496	0.59176	0.58609
Robusta	0.53932	0.84736	0.54281	0.55831
EPO	0.42599	0.68423	0.42361	0.44608
GenomeMatch-3	0.25084	0.45095	0.25491	0.00096
GenomeMatch-1	0.24999	0.45004	0.25423	0.001
GenomeMatch-2	0.24991	0.45201	0.25398	0.00095
Mugsy	0.16379	0.34433	0.15561	0.25913

Table S7: Mammal simulation F-score results stratified by annotation type. Values reported here include self-alignment pairs, that is to say pairs where a sequence is aligned to itself. Rows are sorted in descending order according to overall F-score. Columns are left to right: the F-score of the submission for the entire genome, the F-score of the submission for just genic regions, the F-score of the submission for just the neutral regions and the F-score of the submission for just the repetitive regions.

Name	Overall F-score	Genic F-score	Neutral F-score	Repeats F-score
Mugsy	0.98865	0.99105	0.99144	0.97202
PSAR-Align	0.98607	0.99421	0.98978	0.97409
TBA	0.98499	0.99438	0.98999	0.95677
MULTIZ	0.98469	0.99411	0.98853	0.97125
progressiveMauze	0.98442	0.99172	0.98805	0.97554
AutoMZ	0.98437	0.99412	0.98945	0.95531
Cactus	0.97992	0.99386	0.98279	0.98266
VISTA-LAGAN	0.97836	0.99247	0.98385	0.97185
Pecan	0.97305	0.99417	0.97562	0.9693
Robusta	0.96754	0.99377	0.97104	0.96955
GenomeMatch-1	0.94823	0.98668	0.98836	0.14132
GenomeMatch-2	0.93681	0.98684	0.97807	0.14227
GenomeMatch-3	0.40200	0.98565	0.39074	0.16307

Table S8: Primate simulation F-score results stratified by annotation type. Values reported here include self-alignment pairs, that is to say pairs where a sequence is aligned to itself.

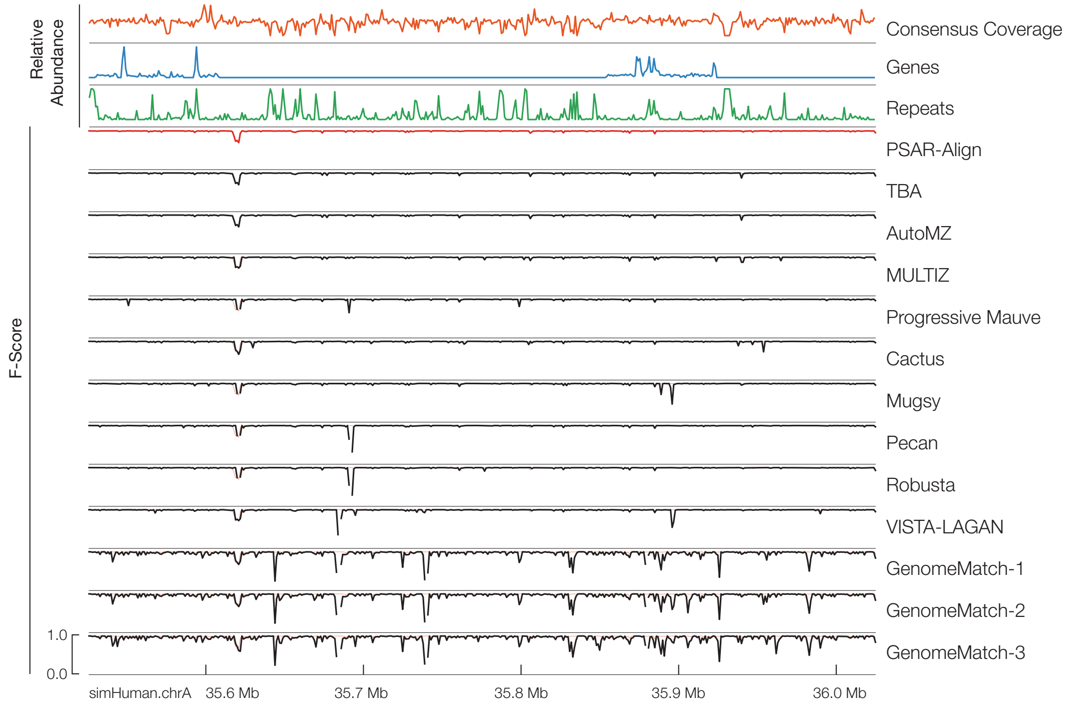

Figure S1: Region 1 of simHuman with respect to simOrang of the regional analysis of the primate simulation data set. Region 1 is defined as bases 35,525,375 –stop 36,025,374 of simHuman chromosome A (horizontal axis). Rows are: the relative true coverage of any part of simMouse onto this region of the reference; the relative abundance of genes within the region; the relative abundance of repetitive sequence in the region; submissions in descending order of average F-score. Each submission row shows the F-score of the submission at a given location of the region in red. The vertical axis of each row is the same scale, as labeled in the bottom row. In grey, in the background, is shown the top submission (PSAR-Align). Note that all of the aligners perform well in this region and that the top ten are likely all within sampling noise of one-another, though the GenomeMatch submissions seem to have a systematic difficulty.

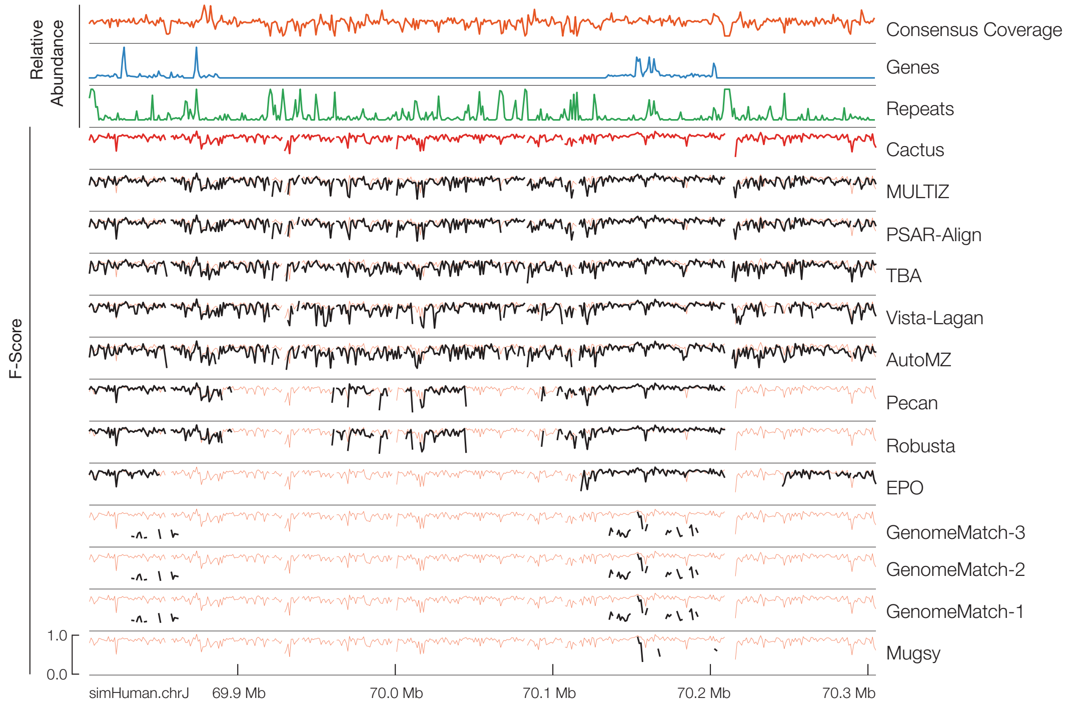

Figure S2: Region 4 of simHuman with respect to simMouse of the regional analysis of the mammal simulation data set. Region 4 is defined as bases 69,805,407 - 70,305,406 of simHuman chromosome J (horizontal axis). Rows are: the relative true coverage of any part of simMouse onto this region of the reference; the relative abundance of genes within the region; the relative abundance of repetitive sequence in the region; submissions in descending order of average F-score. Each submission row shows the F-score of the submission at a given location of the region in black. The vertical axis of each row is the same scale, as labeled in the bottom row. In grey, in the background, is shown the top submission (Cactus). Note that most submissions managed to contain parts of the alignment within the gene regions, though half of the submissions had poor coverage in this region (they lack a black line through most of the plot). The lack of consistent signal from all of the GenomeMatch submissions may be explained by the fact that the submitters excluded repetitive regions from their alignment.

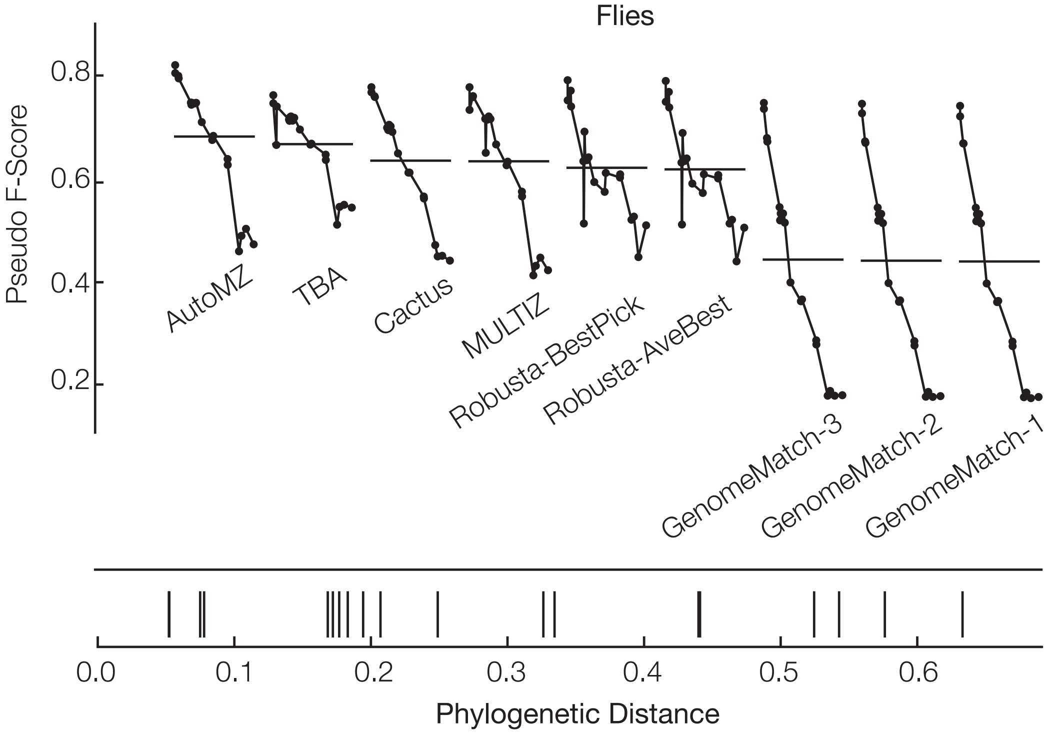

Figure S3: Pseudo F-score results stratified by phylogenetic distance for real fly data set. For each subplot the vertical axis shows the pseudo F-score and the horizontal axis shows individual submissions. Horizontal black lines show the average pseudo F-score of the submission. Submissions are ordered from left to right (descending) by average pseudo F-Score. Submissions are comprised of points connected by a line where the points are in order of phylogenetic distance (relative to the reference, dm3) and vertical lines are $\pm$ standard deviation. The phylogenetic distances of pairs are in ascending left to right order droSec1, droSim1, droYak2, droEre2, droTak, droRho, droBia, droEle, droEug, droFic, droKik, droBip, droAna3, droPer1, droMoj3, droVir3, droWil1, droGri2. For each phylogenetic pair point in the figure, the recall value is taken to be the average within region pairwise coverage.

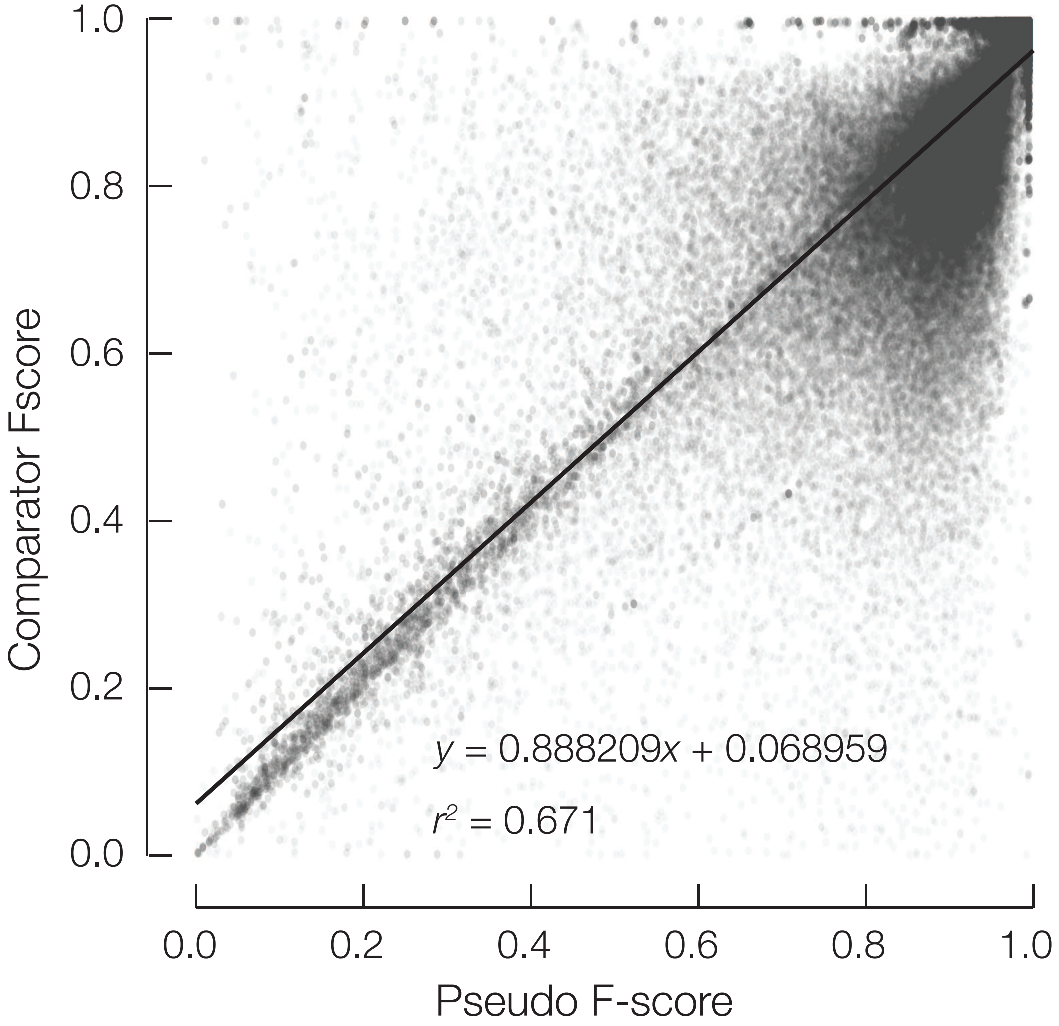

Figure S4: Correlation between PSAR-based pseudo-F-Scores and F-Scores for identical bins with respect to the reference (simHuman for both the simulated Primate and simulated Mammalian data sets) using all pairs of species that include the reference and derived from all submissions. Transparency ($\alpha = 0.05$) is used to represent the data points ($n = 174,110$) such that highly dense areas are darker than others. The strong cloud of data points to the upper right are bins and pairs derived almost entirely from the simulated Primate data set. The overall trend is roughly linear with a coefficient of determination $r^2 = 0.671$.

Name	Pseudo F-score Region mean	Pseudo F-score Region std
MULTIZ	0.659	3.74e-04
Cactus	0.656	5.27e-04
AutoMZ	0.651	3.93e-04
TBA	0.620	5.93e-04
Robusta-BestPick	0.590	8.58e-04
Robusta-AveBest	0.585	7.63e-04
GenomeMatch-1	0.399	8.59e-05
GenomeMatch-3	0.399	9.73e-05
GenomeMatch-2	0.396	8.95e-05

Table S9: Fly pseudo F-score regional results. Rows are ordered by descending value of pseudo F-score region mean value. Columns are: pseudo F-score region mean; pseudo F-score region standard deviation. Values are the means and standard deviations of the submission within the five regions, with pseudo F-score calculated using the overall average coverage for all pairs of species in the submission as the recall value.

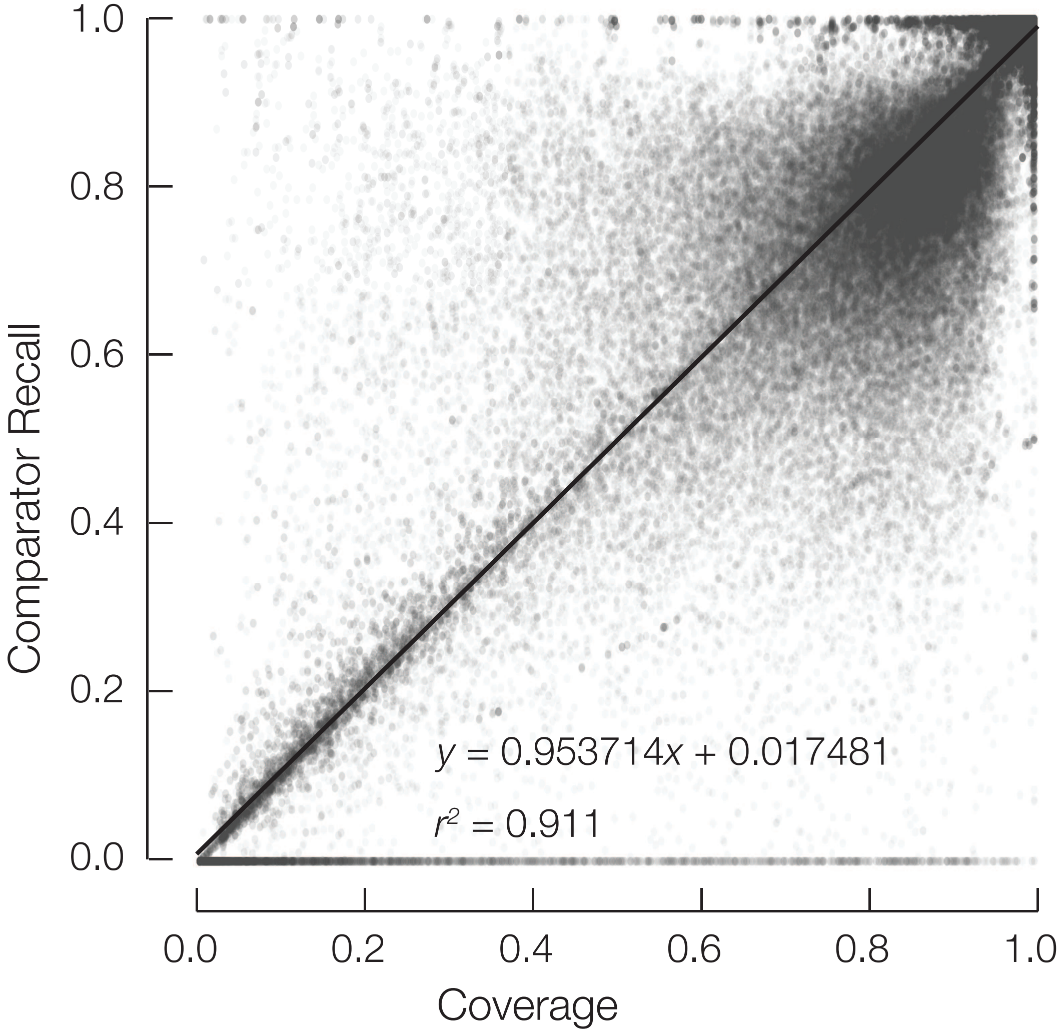

Figure S5: Correlation between coverage and recall for identical bins with respect to the reference (simHuman for both the simulated Primate and simulated Mammal data sets) using all pairs of species that include the reference and derived from all submissions. Data points ($n = 216,536$) are slightly transparent ($\alpha = 0.05$) which allows the viewer to get a sense for the density of data points in some areas relative to others. There were 10,964 bins that were omitted due to not having a recall value. The strong cloud of data points to the upper right are bins and pairs derived almost entirely from the simulated Primate data set. The overall trend is linear with a coefficient of determination $r^2 = 0.911$.

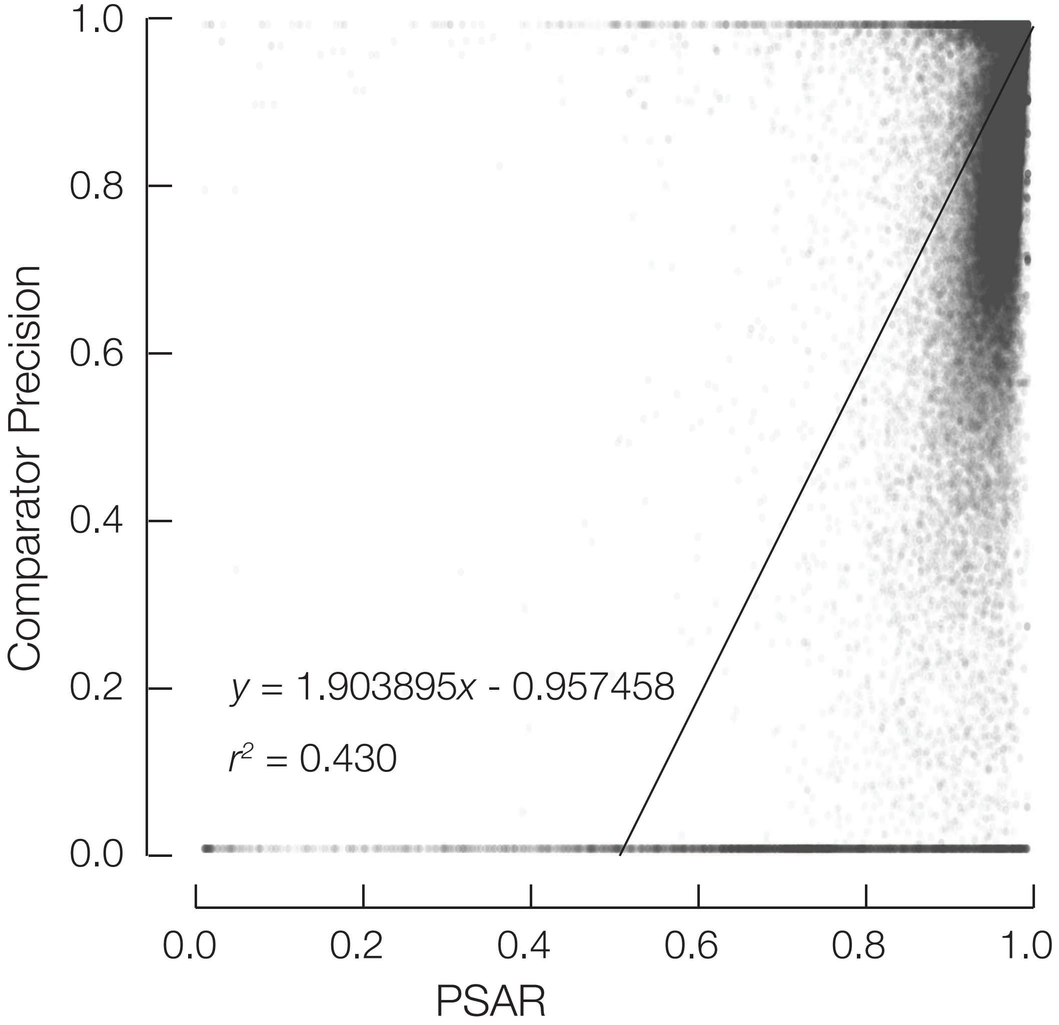

Figure S6: Correlation between PSAR precision and precision for identical bins with respect to the reference (simHuman for both the simulated Primate and simulated Mammal data sets) using all pairs of species that include the reference and derived from all submissions. Data points ($n = 178,007$) are slightly transparent ($\alpha = 0.05$) which allows the viewer to get a sense for the density of data points in some areas relative to others. The cloud of points in the upper right indicates very high PSAR-precision values with precision values ranging from very high to moderate. This result is indicative of PSAR-precision not being particularly accurate. The coefficient of determination $r^2 = 0.430$.

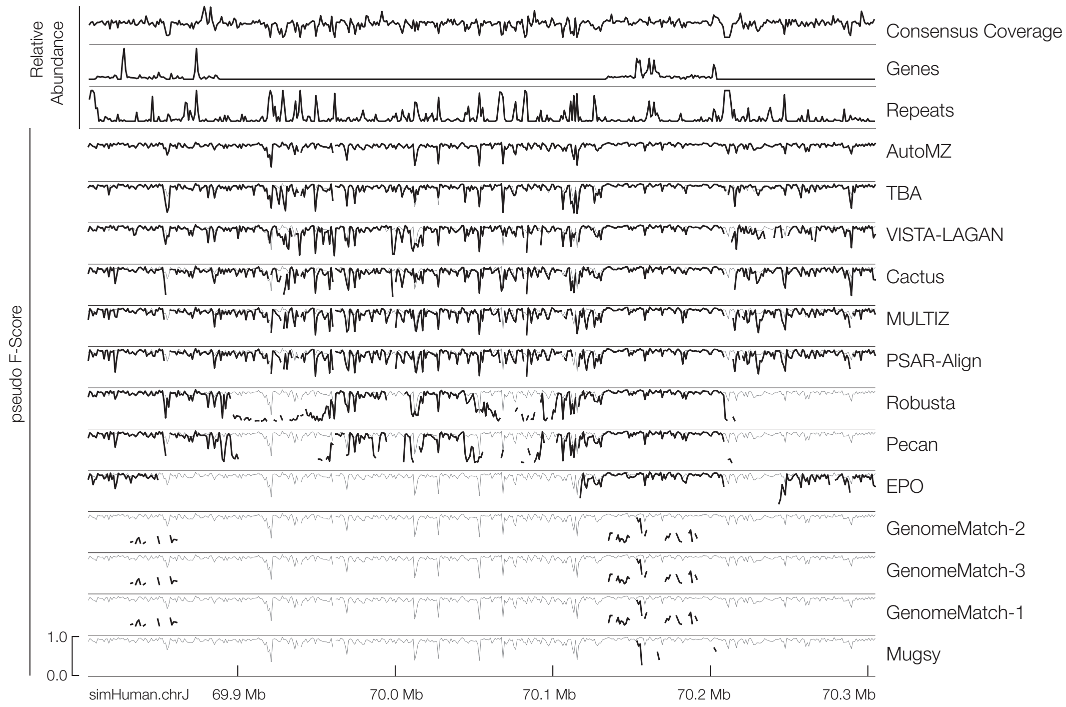

Figure S7: Pseudo F-score along region 4 of simHuman with respect to simMouse of the regional analysis of the mammal simulation data set. Region 4 is defined as bases 69,805,407 - 70,305,406 of simHuman chromosome J (horizontal axis). Rows are: the relative true coverage of any part of simMouse onto this region of the reference; the relative abundance of genes within the region; the relative abundance of repetitive sequence in the region; submissions in descending order of average pseudo F-score. Each submission row shows the pseudo F-score of the submission at a given location of the region in black. The vertical axis of each row is the same scale, as labeled in the bottom row. In grey, in the background, is shown the top submission (AutoMZ). Note that most submissions managed to contain parts of the alignment within the gene regions, though half of the submissions had poor coverage in this region (they lack a black line through most of the plot).

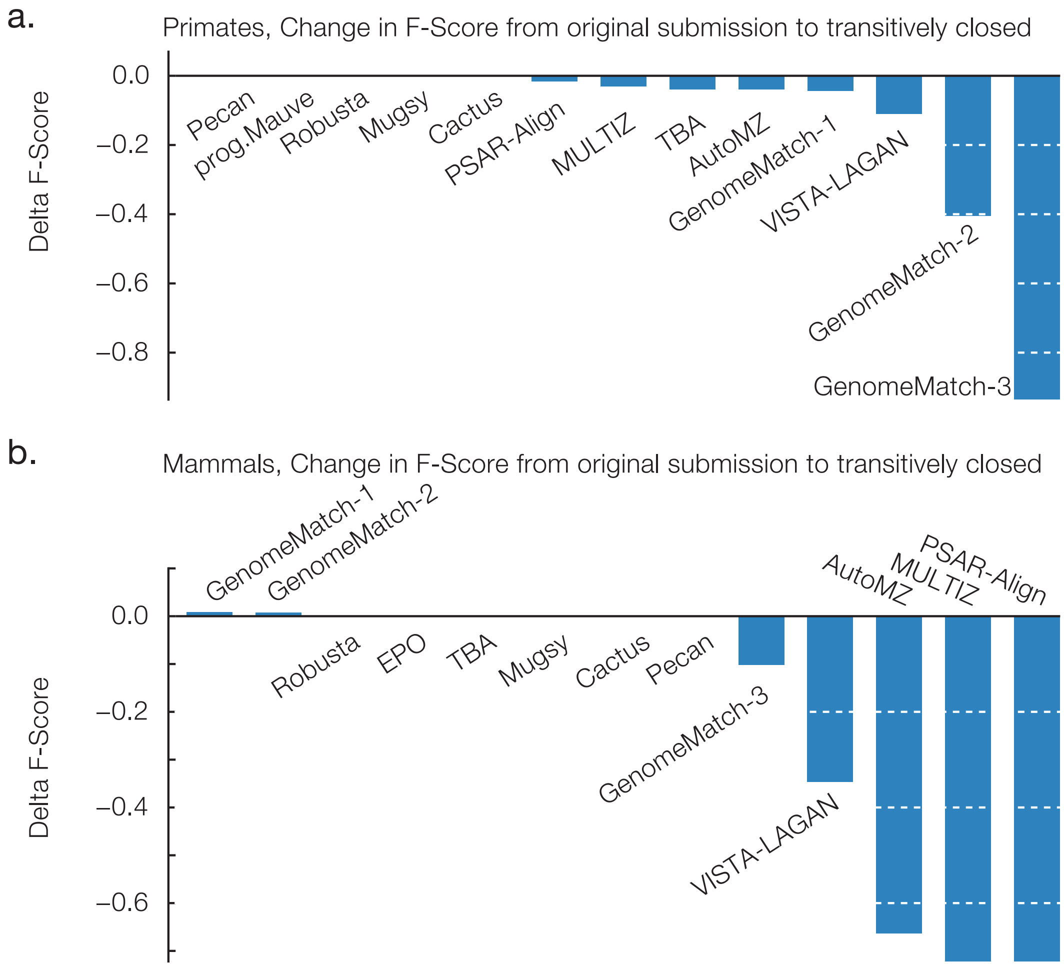

Figure S8: The change in F-Score between original submissions (exactly as provided by participants) and the transitive closure of those same alignments for the simulated primate (A) and simulated mammal (B) data sets. A negative value indicates that following transitive closure the alignment had a lower F-Score.

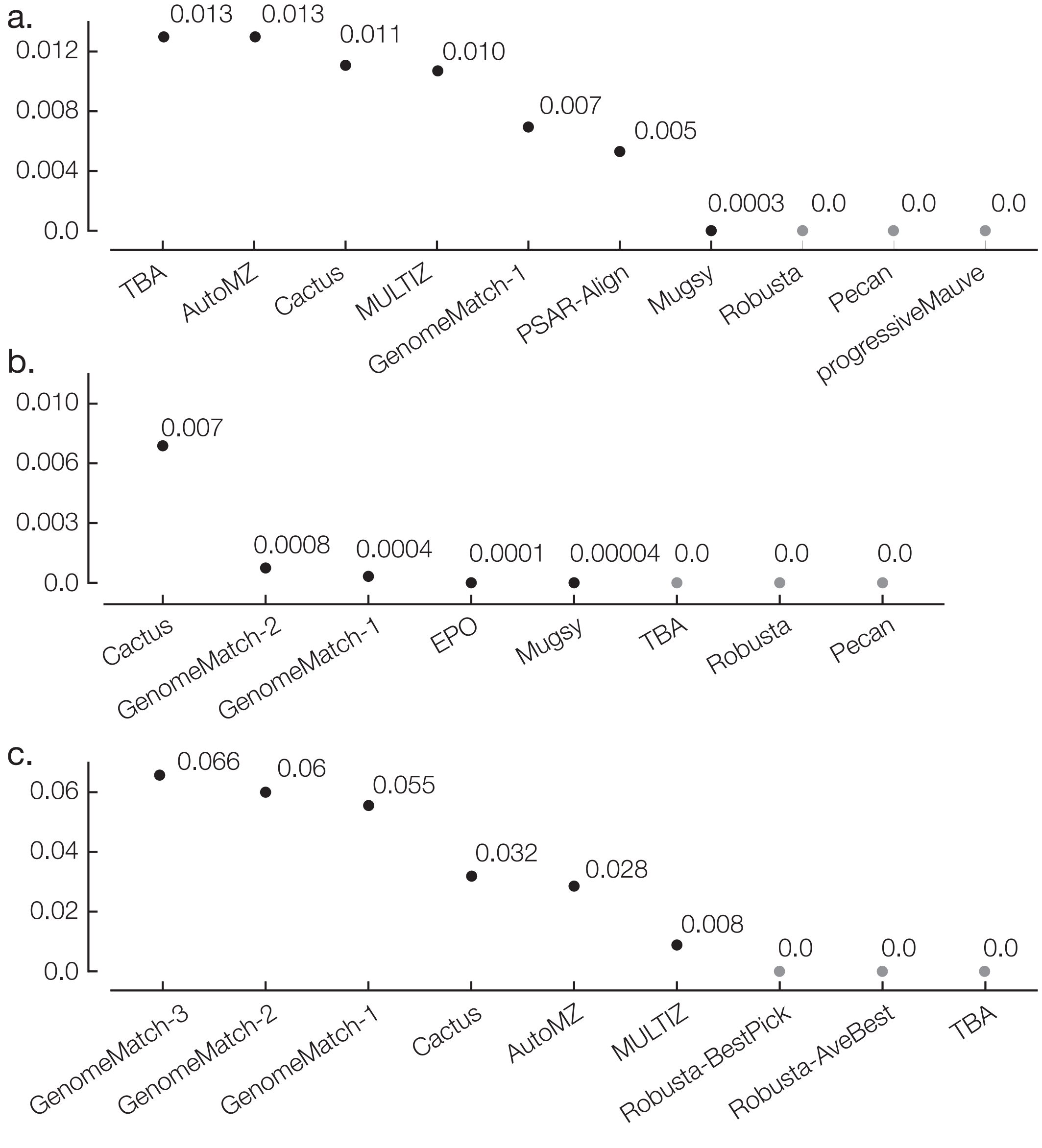

Figure S9: The overall average proportion of duplications for transitively closed submissions for (A) the primate dataset, (B) the mammal dataset and (C) the fly dataset of the transitively closed submissions. For A and B, only submissions that were not affected by transitive closure (see Figure S8) by more than 0.05 are shown. All submissions for flies are shown in part C. Gray data points are used to indicate 0 values at the 10^{-6} level.

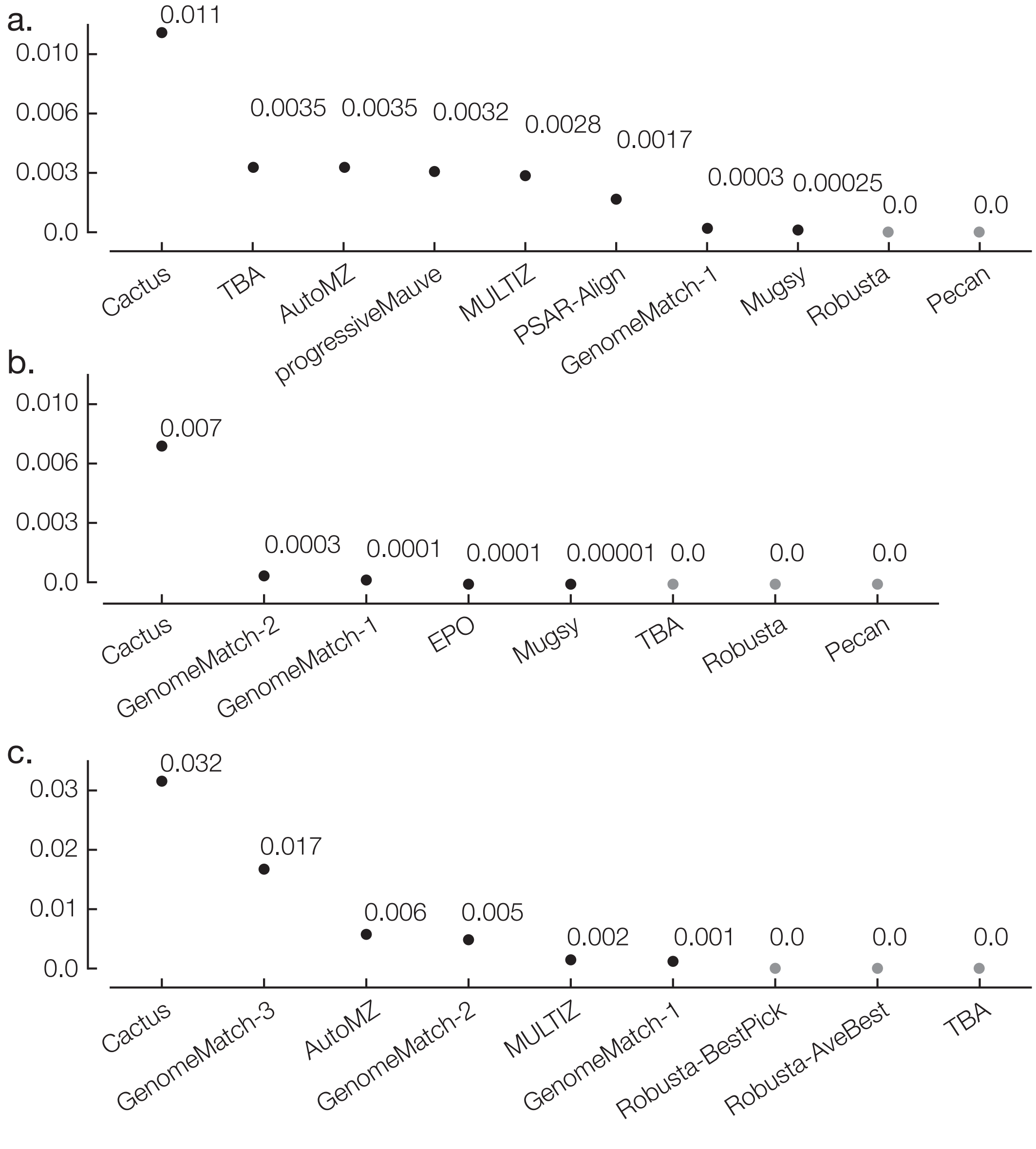

Figure S10: The overall average proportion of duplications for original submissions for (A) the primate dataset, (B) the mammal dataset and (C) the fly dataset of the transitively closed submissions. For A and B, only submissions that were not affected by transitive closure (see Figure S8) by more than 0.05 are shown. All submissions for flies are shown in part C. Gray data points are used to indicate 0 values at the 10^{-6} level.

-	GM-3	GM-2	GM-1	Robusta	Pecan	Cactus	V-L	pM	Mugsy	TBA	AutoMz	Multiz	PSAR-A	Truth
GenomeMatch-3	0	0.74307	0.74908	0.7646	0.76469	0.75208	0.75172	0.75679	0.75514	0.75427	0.75434	0.75432	0.75412	0.75437
GenomeMatch-2	0.74307	0	0.02477	0.15127	0.14458	0.12562	0.13205	0.11593	0.11313	0.11646	0.11687	0.11629	0.11294	0.12413
GenomeMatch-1	0.74908	0.02477	0	0.13156	0.12475	0.10788	0.11653	0.09564	0.0929	0.0972	0.09771	0.09679	0.09367	0.10408
Robusta	0.7646	0.15127	0.13156	0	0.02293	0.07713	0.07859	0.05982	0.06503	0.06622	0.0672	0.06456	0.06388	0.07149
EBI-MP	0.76469	0.14458	0.12475	0.02293	0	0.06808	0.07276	0.05449	0.05585	0.06034	0.06117	0.05918	0.05779	0.06352
Cactus	0.75208	0.12562	0.10788	0.07713	0.06808	0	0.04163	0.0393	0.03374	0.03482	0.03589	0.03505	0.0325	0.03276
VISTA-LAGAN	0.75172	0.13205	0.11653	0.07859	0.07276	0.04163	0	0.03734	0.03666	0.03229	0.03315	0.03216	0.02955	0.03824
progressiveMauve	0.75679	0.11593	0.09564	0.05982	0.05449	0.0393	0.03734	0	0.0257	0.02412	0.02509	0.02331	0.02157	0.03174
Mugsy	0.75514	0.11313	0.0929	0.06503	0.05585	0.03374	0.03666	0.0257	0	0.02301	0.02393	0.02355	0.02058	0.02603
TBA	0.75427	0.11646	0.0972	0.06622	0.06034	0.03482	0.03229	0.02412	0.02301	0	0.00148	0.0158	0.01099	0.02658
AutoMZ	0.75434	0.11687	0.09771	0.0672	0.06117	0.03589	0.03315	0.02509	0.02393	0.00148	0	0.01462	0.0096	0.02765
MULTIZ	0.75432	0.11629	0.09679	0.06456	0.05918	0.03505	0.03216	0.02331	0.02355	0.0158	0.01462	0	0.00893	0.0274
PSAR-Align	0.75412	0.11294	0.09367	0.06388	0.05779	0.0325	0.02955	0.02157	0.02058	0.01099	0.0096	0.00893	0	0.02451
Truth	0.75437	0.12413	0.10408	0.07149	0.06352	0.03276	0.03824	0.03174	0.02603	0.02658	0.02765	0.0274	0.02451	0

Table S10: Jaccard distance values for all submissions to the primate dataset.

-	GM-3	GM-2	GM-1	Mugsy	Robusta	Pecan	EPO	TBA	AutoMz	Multiz	PSAR-A	V-L	Cactus	Truth
GenomeMatch-3	0	0.01602	0.02215	0.56734	0.84947	0.81879	0.80496	0.87589	0.90189	0.88911	0.88794	0.87452	0.86813	0.89433
GenomeMatch-2	0.01602	0	0.00638	0.56642	0.85082	0.82031	0.80608	0.87764	0.90328	0.89056	0.88921	0.87679	0.87029	0.89642
GenomeMatch-1	0.02215	0.00638	0	0.56471	0.85052	0.82004	0.80546	0.87774	0.90343	0.89068	0.88955	0.87759	0.87016	0.89635
Mugsy	0.56734	0.56642	0.56471	0	0.90826	0.88851	0.86479	0.92279	0.94182	0.93541	0.93471	0.92353	0.91915	0.93515
Robusta	0.84947	0.85082	0.85052	0.90826	0	0.33374	0.65927	0.66245	0.70596	0.66041	0.65686	0.65332	0.62052	0.68229
EBI-MP	0.81879	0.82031	0.82004	0.88851	0.33374	0	0.57069	0.62906	0.67906	0.62515	0.62073	0.6167	0.56893	0.64957
EBI-EPO	0.80496	0.80608	0.80546	0.86479	0.65927	0.57069	0	0.76112	0.795	0.76115	0.75807	0.7538	0.72852	0.78167
TBA	0.87589	0.87764	0.87774	0.92279	0.66245	0.62906	0.76112	0	0.37011	0.33682	0.33147	0.3923	0.3803	0.4633
AutoMZ	0.90189	0.90328	0.90343	0.94182	0.70596	0.67906	0.795	0.37011	0	0.30333	0.29905	0.46523	0.45694	0.52181
MULTIZ	0.88911	0.89056	0.89068	0.93541	0.66041	0.62515	0.76115	0.33682	0.30333	0	0.0311	0.39263	0.37148	0.45769
PSAR-Align	0.88794	0.88921	0.88955	0.93471	0.65686	0.62073	0.75807	0.33147	0.29905	0.0311	0	0.3879	0.3658	0.45749
VISTA-LAGAN	0.87452	0.87679	0.87759	0.92353	0.65332	0.6167	0.7538	0.3923	0.46523	0.39263	0.3879	0	0.37819	0.46559
Cactus	0.86813	0.87029	0.87016	0.91915	0.62052	0.56893	0.72852	0.3803	0.45694	0.37148	0.3658	0.37819	0	0.35431
Truth	0.89433	0.89642	0.89635	0.93515	0.68229	0.64957	0.78167	0.4633	0.52181	0.45769	0.45749	0.46559	0.35431	0

Table S11: Jaccard distance values for all submissions to the mammal dataset.

-	GM-3	GM-2	GM-1	R-BestPick	R-AveBest	Cactus	TBA	Multiz	AutoMz
GenomeMatch-3	0	0.42102	0.40979	0.64743	0.64402	0.60489	0.71506	0.56541	0.60301
GenomeMatch-2	0.42102	0	0.09748	0.60592	0.60134	0.57177	0.60991	0.51772	0.55361
GenomeMatch-1	0.40979	0.09748	0	0.60166	0.59743	0.57011	0.56577	0.51316	0.54323
Robusta-BestPick	0.64743	0.60592	0.60166	0	0.26152	0.60582	0.58221	0.55836	0.57614
Robusta-AveBest	0.64402	0.60134	0.59743	0.26152	0	0.59518	0.56673	0.54312	0.56131
Cactus	0.60489	0.57177	0.57011	0.60582	0.59518	0	0.51964	0.48787	0.50365
TBA	0.71506	0.60991	0.56577	0.58221	0.56673	0.51964	0	0.3587	0.32854
MULTIZ	0.56541	0.51772	0.51316	0.55836	0.54312	0.48787	0.3587	0	0.25761
AutoMZ	0.60301	0.55361	0.54323	0.57614	0.56131	0.50365	0.32854	0.25761	0

Table S12: Jaccard distance values for all submissions to the fly dataset.

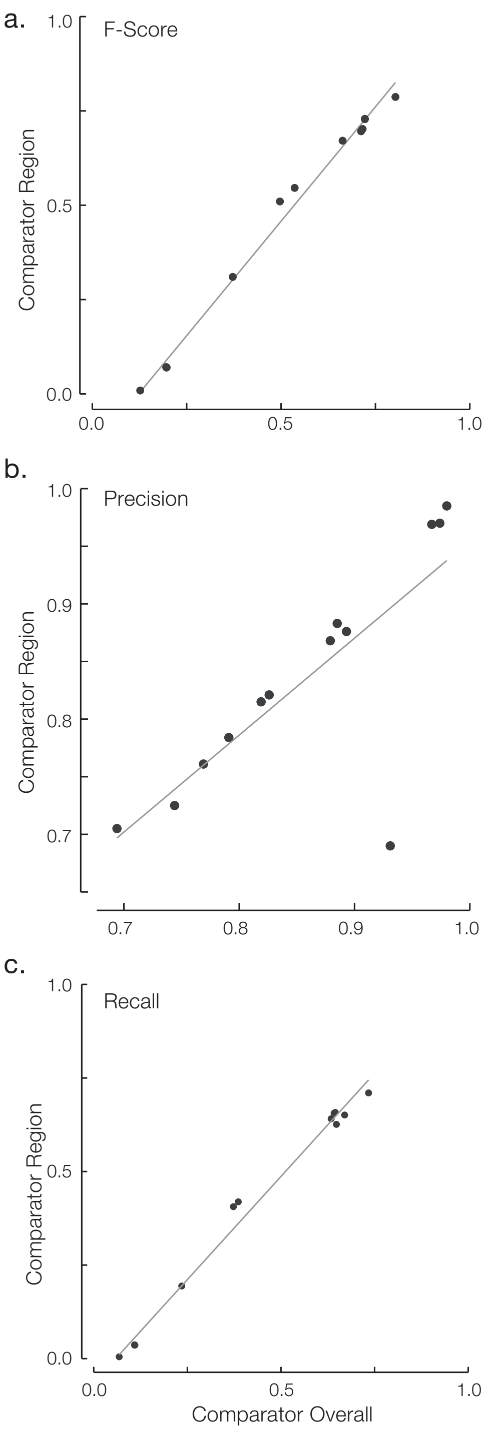

Figure S11: Correlation plots of comparator values between the regional and whole genome analyses.

Name	Precision Overall	Precision Region mean	Precision Region std	Precision Region mean	Precision Region std
Progressive_Mauve	0.997	0.997	0.000437	0.998	0.00052
GenomeMatch-1	0.997	0.997	0.000292	0.998	0.000368
Pecan	0.996	0.997	0.00143	0.995	0.0049
Mugsy	0.996	0.997	0.000623	0.999	0.000357
PSAR-Align	0.995	0.998	0.000489	0.999	0.000134
MULTIZ	0.992	0.995	0.000964	0.998	0.00022
AutoMZ	0.992	0.996	0.00134	0.999	0.000162
TBA	0.992	0.996	0.00135	0.999	0.000172
Cactus	0.986	0.996	0.000426	0.999	0.0000821
Robusta	0.986	0.99	0.00764	0.992	0.00771
VISTA-LAGAN	0.983	0.995	0.00144	0.998	0.000299
GenomeMatch-2	0.972	0.996	0.00164	0.998	0.000704
GenomeMatch-3	0.261	0.985	0.00244	0.994	0.000754

Table S13: Primate simulation precision results. Rows are ordered by descending value of overall (full genome) precision. Columns are: precision overall value; precision region mean; precision region standard deviation; precision region mean; precision region standard deviation.

Name	Recall Overall	Recall Region mean	Recall Region std	Recall Region mean	Recall Region std
Cactus	0.987	0.978	0.00328	1	0.000667
TBA	0.986	0.984	0.00372	1	0.000333
AutoMZ	0.984	0.984	0.00372	1	0.000333
MULTIZ	0.984	0.984	0.00397	1	0
PSAR-Align	0.984	0.983	0.0041	0.999	0.00126
Mugsy	0.982	0.982	0.00412	1	0.000385
VISTA-LAGAN	0.982	0.976	0.0059	0.999	0.00115
Progressive_Mauve	0.975	0.977	0.00134	0.998	0.00213
Robusta	0.945	0.97	0.0146	1	0.000385
Pecan	0.944	0.97	0.0146	0.997	0.00255
GenomeMatch-3	0.908	0.876	0.00292	0.989	0.00418
GenomeMatch-1	0.902	0.906	0.00629	0.988	0.00394
GenomeMatch-2	0.902	0.901	0.00677	0.988	0.00394

Table S14: Primate simulation recall results. Rows are ordered by descending value of overall (full genome) recall. Columns are: recall overall value; recall region mean; recall region standard deviation; recall region mean; recall region standard deviation.

Name	Precision Overall	Precision Region mean	Precision Region std	Precision Region mean	Precision Region std
GenomeMatch-1	0.98	0.985	0.00216	0.92	0.0153
GenomeMatch-2	0.974	0.97	0.0298	0.917	0.0172
GenomeMatch-3	0.967	0.969	0.0307	0.916	0.0174
Mugsy	0.931	0.69	0.319	0.949	0.0309
EBI-EPO	0.893	0.876	0.0264	0.925	0.0722
Cactus	0.885	0.883	0.00433	0.974	0.00196
EBI-MP	0.879	0.868	0.00701	0.864	0.154
PSAR-Align	0.826	0.821	0.0065	0.972	0.00183
MULTIZ	0.819	0.815	0.00735	0.971	0.00177
VISTA-LAGAN	0.791	0.784	0.0111	0.959	0.000299
TBA	0.769	0.761	0.00959	0.964	0.00158
Robusta	0.744	0.725	0.074	0.849	0.134
AutoMZ	0.694	0.705	0.0204	0.955	0.00352

Table S15: Mammal simulation precision results. Rows are ordered by descending value of overall (full genome) precision. Columns are: precision overall value; precision region mean; precision region standard deviation; precision region mean; precision region standard deviation.

Name	Recall Overall	Recall Region mean	Recall Region std	Recall Region mean	Recall Region std
Cactus	0.734	0.71	0.0166	0.977	0.00537
TBA	0.67	0.651	0.0117	0.994	0.00155
VISTA-LAGAN	0.648	0.626	0.00979	0.979	0.00575
MULTIZ	0.645	0.658	0.016	0.975	0.00667
PSAR-Align	0.642	0.656	0.0164	0.973	0.00633
AutoMZ	0.634	0.641	0.013	0.997	0.00286
EBI-MP	0.386	0.419	0.203	0.681	0.207
Robusta	0.373	0.406	0.186	0.785	0.155
EBI-EPO	0.235	0.194	0.0933	0.366	0.0509
GenomeMatch-3	0.11	0.036	0.0144	0.393	0.281
GenomeMatch-1	0.109	0.037	0.0144	0.392	0.282
GenomeMatch-2	0.109	0.036	0.0144	0.393	0.282
Mugsy	0.068	0.005	0.00404	0.144	0.238

Table S16: Mammal simulation recall results. Rows are ordered by descending value of overall (full genome) recall. Columns are: recall overall value; recall region mean; recall region standard deviation; recall region mean; recall region standard deviation.

Name	Overall Precision	Genic Precision	Neutral Precision	Repeats Precision
GenomeMatch-1	0.84785	0.86579	0.84681	0.88833
GenomeMatch-2	0.84258	0.86509	0.84281	0.88745
GenomeMatch-3	0.83411	0.86554	0.83464	0.85542
Mugsy	0.79971	0.86068	0.82456	0.46825
EPO	0.7326	0.82595	0.74124	0.65269
Pecan	0.72711	0.82935	0.73811	0.63312
Cactus	0.64093	0.81672	0.64769	0.59468
Robusta	0.61421	0.82501	0.62934	0.59633
PSAR-Align	0.60382	0.81435	0.61773	0.33265
MULTIZ	0.59694	0.81411	0.61057	0.32802
VISTA-LAGAN	0.58672	0.81773	0.6013	0.50301
TBA	0.56616	0.81574	0.58856	0.27129
AutoMZ	0.50609	0.81298	0.5308	0.14208

Table S17: Mammal simulation precision results stratified by annotation type. Values reported here include self-alignment pairs, that is to say pairs where a sequence is aligned to itself. Rows are sorted in descending order according to overall precision. Columns are left to right: the precision of the submission for the entire genome, the precision of the submission for just genic regions, the precision of the submission for just the neutral regions and the precision of the submission for just the repetitive regions.

Name	Overall Recall	Genic Recall	Neutral Recall	Repeats Recall
Cactus	0.83312	0.92573	0.83822	0.76319
TBA	0.76887	0.896	0.77371	0.67603
VISTA-LAGAN	0.74612	0.89397	0.75053	0.6307
MULTIZ	0.73313	0.89601	0.74203	0.39679
PSAR-Align	0.73113	0.89595	0.73966	0.39941
AutoMZ	0.71996	0.88765	0.72877	0.37684
Pecan	0.49704	0.8822	0.49385	0.54556
Robusta	0.48072	0.87096	0.4772	0.52485
EPO	0.3003	0.58402	0.29654	0.33882
GenomeMatch-3	0.14762	0.3049	0.15042	0.00048
GenomeMatch-2	0.14671	0.30593	0.14952	0.00047
GenomeMatch-1	0.14661	0.30404	0.14957	0.0005
Mugsy	0.09124	0.21521	0.08591	0.17913

Table S18: Mammal simulation recall results stratified by annotation type. Values reported here include self-alignment pairs, that is to say pairs where a sequence is aligned to itself. Rows are sorted in descending order according to overall recall. Columns are left to right: the recall of the submission for the entire genome, the recall of the submission for just genic regions, the recall of the submission for just the neutral regions and the recall of the submission for just the repetitive regions.

Name	Overall Precision	Genic Precision	Neutral Precision	Repeats Precision
Pecan	0.98865	0.992	0.99146	0.98278
GenomeMatch-1	0.98616	0.99162	0.9865	0.99018
Progressive_Mauve	0.98445	0.99186	0.98784	0.97814
Mugsy	0.98424	0.99186	0.98713	0.96651
PSAR-Align	0.98041	0.99201	0.98406	0.97261
MULTIZ	0.97754	0.99189	0.98213	0.95795
AutoMZ	0.97686	0.99191	0.98369	0.93208
TBA	0.97678	0.99188	0.98357	0.93148
Robusta	0.97675	0.992	0.98087	0.98159
VISTA-LAGAN	0.96793	0.99254	0.97546	0.96921
Cactus	0.96627	0.99141	0.96969	0.97344
GenomeMatch-2	0.9618	0.99169	0.96621	0.9884
GenomeMatch-3	0.25751	0.98859	0.24326	0.98011

Table S19: Primate simulation precision results stratified by annotation type. Values reported here include self-alignment pairs, that is to say pairs where a sequence is aligned to itself. Rows are sorted in descending order according to overall precision. Columns are left to right: the precision of the submission for the entire genome, the precision of the submission for just genic regions, the precision of the submission for just the neutral regions and the precision of the submission for just the repetitive regions.

Name	Overall Recall	Genic Recall	Neutral Recall	Repeats Recall
Cactus	0.99396	0.99631	0.99624	0.99205
TBA	0.99334	0.99689	0.99648	0.98347
Mugsy	0.99309	0.99024	0.99579	0.9776
AutoMZ	0.992	0.99634	0.99527	0.97973
MULTIZ	0.99194	0.99634	0.99501	0.98492
PSAR-Align	0.99179	0.99642	0.99556	0.97557
VISTA-LAGAN	0.98902	0.9924	0.99239	0.9745
Progressive_Mauve	0.98438	0.99157	0.98827	0.97296
Robusta	0.95851	0.99554	0.96139	0.9578
Pecan	0.95794	0.99635	0.96028	0.95618
GenomeMatch-3	0.91598	0.98273	0.99231	0.08893
GenomeMatch-1	0.91312	0.98179	0.99022	0.07609
GenomeMatch-2	0.91308	0.98203	0.99023	0.07665

Table S20: Primate simulation recall results stratified by annotation type. Values reported here include self-alignment pairs, that is to say pairs where a sequence is aligned to itself. Rows are sorted in descending order according to overall recall. Columns are left to right: the recall of the submission for the entire genome, the recall of the submission for just genic regions, the recall of the submission for just the neutral regions and the recall of the submission for just the repetitive regions.

Bibliography

Blanchette, M., Kent, W. J., Riemer, C., Elnitski, L., Smit, A. F. A., Roskin, K. M., Baertsch, R., Rosenbloom, K., Clawson, H., Green, E. D., et al., 2004. Aligning multiple genomic sequences with the threaded blockset aligner. *Genome Research* **14**:708–715.

Brudno, M., Malde, S., Poliakov, A., Do, C. B., Couronne, O., Dubchak, I., & Batzoglou, S., 2003. Glocal alignment: finding rearrangements during alignment. *Bioinformatics (Oxford, England)* **19 Suppl 1**:i54–62.

Darling, A. E., Mau, B., & Perna, N. T., 2010. progressiveMauve: multiple genome alignment with gene gain, loss and rearrangement. *PLoS ONE* **5**:e11147.

Dubchak, I., Poliakov, A., Kislyuk, A., & Brudno, M., 2009. Multiple whole-genome alignments without a reference organism. *Genome Research* **19**:682–689.

Frazer, K. A., Pachter, L., Poliakov, A., Rubin, E. M., & Dubchak, I., 2004. VISTA: computational tools for comparative genomics. *Nucleic Acids Research* **32**:W273–W279.

Kent, W. J., 2002. BLAT—The BLAST-Like Alignment Tool. *Genome Research* **12**:656–664.

Kent, W. J., Baertsch, R., Hinrichs, A., Miller, W., & Haussler, D., 2003. Evolution's cauldron: duplication, deletion, and rearrangement in the mouse and human genomes. *Proceedings of the National Academy of Sciences of the United States of America* **100**:11484–11489.

Paten, B., Earl, D., Nguyen, N., Diekhans, M., Zerbino, D., & Haussler, D., 2011. Cactus: Algorithms for genome multiple sequence alignment. *Genome Research* **21**:1512–1528.
